# Conversational Interaction in the Scanner: Mentalizing during Language Processing as Revealed by MEG

**DOI:** 10.1093/cercor/bhu116

**Published:** 2014-06-05

**Authors:** Sara Bögels, Dale J. Barr, Simon Garrod, Klaus Kessler

**Affiliations:** 1 Max Planck Institute for Psycholinguistics, Nijmegen, The Netherlands; 2 Institute of Neuroscience and Psychology, University of Glasgow, Glasgow, UK; 3 Aston Brain Centre, Aston University, Birmingham, UK

**Keywords:** conversational interaction, episodic working memory, magnetoencephalography, perspective taking, pragmatics, theory of mind

## Abstract

Humans are especially good at taking another's perspective—representing what others might be thinking or experiencing. This “mentalizing” capacity is apparent in everyday human interactions and conversations. We investigated its neural basis using magnetoencephalography. We focused on whether mentalizing was engaged spontaneously and routinely to understand an utterance's meaning or largely on-demand, to restore “common ground” when expectations were violated. Participants conversed with 1 of 2 confederate speakers and established tacit agreements about objects' names. In a subsequent “test” phase, some of these agreements were violated by either the same or a different speaker. Our analysis of the neural processing of test phase utterances revealed recruitment of neural circuits associated with language (temporal cortex), episodic memory (e.g., medial temporal lobe), and mentalizing (temporo-parietal junction and ventromedial prefrontal cortex). Theta oscillations (3–7 Hz) were modulated most prominently, and we observed phase coupling between functionally distinct neural circuits. The episodic memory and language circuits were recruited in anticipation of upcoming referring expressions, suggesting that context-sensitive predictions were spontaneously generated. In contrast, the mentalizing areas were recruited on-demand, as a means for detecting and resolving perceived pragmatic anomalies, with little evidence they were activated to make partner-specific predictions about upcoming linguistic utterances.

## Introduction

In conversation, the meaning of linguistic expressions such as “the red couch” is often ambiguous, such that interlocutors need to work together to make sure their interpretations are aligned (Pickering and Garrod 2004). One influential proposal assumes that interlocutors align their interpretations by processing language against their “common ground,” the set of mutual beliefs and expectations that are shared, and critically, “known to be shared” with their interlocutors (Lewis 1979; Clark and Marshall 1981; Stalnaker 1987). This collaborative model of conversation assumes that the memory representations forming the common ground are built up through a process of “grounding” (Clark and Brennan 1991). Processing language consistently with common ground involves accessing representations that are known to be shared, and suppressing information known privately to oneself.

The process of inferring another's mental states, or “mentalizing” (e.g., Frith and Frith 2006) is likely to be involved not only in establishing common ground representations but also in selectively accessing and maintaining such representations in a context-sensitive manner. Language users cannot always count on others having the same perceptual states and experiences and so must on occasion modulate what information they use in speaking and understanding to be consistent with what they know about their interlocutor. Behavioral evidence suggests that during conversation, mentalizing is often called upon for resolving referential ambiguity, especially in cases in which there are clear differences in perspective (Keysar et al. 2003; Kronmüller and Barr 2007).

Given the inherent ambiguity of language, mentalizing would seem to be an essential ingredient of successful communication (Metzing and Brennan 2003; Kronmüller and Barr 2007). However, research over the past several decades indicates that mentalizing is effortful (Roβnagel 2000; Nilsen and Graham 2009; Lin et al. 2010) and that language users have access to other strategies for resolving ambiguity that do not involve mentalizing (Ferreira and Dell 2000; Pickering and Garrod 2004; Shintel and Keysar 2007; Barr 2014).

One case that is relevant to the current investigation concerns how basic memory processes activate contextually relevant information during conversational language processing. During interaction, the perceptual experiences associated with hearing and seeing a particular interlocutor will tend to increase the accessibility of information in long-term memory that is associated with that interlocutor; in this way, basic memory mechanisms promote contextually appropriate speaking and understanding. However, the episodic representations that are forged through conversational interaction are not identical to common ground, as episodic representations can be shared without also being known to be shared (Shintel and Keysar 2007). Furthermore, contextually appropriate representations may become activated in a contextually appropriate manner via the basic memory retrieval principle of encoding specificity rather than through mentalizing (Barr et al. 2014). Supporting this view, a recent study shows that when a speaker articulates an utterance designed by another speaker (e.g., reading someone else's email aloud), listeners activate information associated with the person delivering the message (the messenger/reader) rather than with the person who designed it, despite the relevant common ground being that which is shared with the utterance designer (Barr et al. 2014).

In short, because processes other than mentalizing can promote successful communication, in any given case in which language users adapt their language processing to context, it is an empirical question whether or not such adaptation involved mentalizing as an indicator of genuine partner-oriented processing. Our study therefore set out to investigate these questions using magnetoencephalography (MEG) to monitor listeners′ unfolding interpretations of referentially ambiguous expressions during live social interaction: We tested whether mentalizing would occupy a central, anticipatory role or a more “on demand” role within common ground processing, which has been of some debate recently (e.g., Metzing and Brennan 2003; Kronmüller and Barr 2007). MEG is well suited to the study of spoken language processing in context, because it provides the necessary temporal and spectral resolution to examine moment-by-moment changes in activation and modulation of neural oscillations, as well as sufficient spatial resolution to enable localization of function and the identification of brain networks.

Previous neuroimaging studies have addressed issues of referential ambiguity, memory, and mentalizing, but usually these issues are addressed separately in different studies, often in non-conversational settings with socially isolated participants. While EEG studies have documented a remarkably early sensitivity to referential ambiguity ("NRef" effect; Van Berkum et al. 1999; Van Berkum et al. 2003), it remains unclear whether such effects can be modulated by conversational memory or by beliefs about common ground, as participants in these studies were presented with prepared text or speech in social isolation. An fMRI study of communicative perspective taking reported activations related to referential ambiguity in the superior dorsal medial prefrontal cortex, bilateral middle temporal gyri, and the left temporal pole, whereas activations in the left precuneus and bilateral temporo-parietal junctions (TPJs) particularly reflected the presence vs. absence of an avatar during referential instructions (Dumontheil et al. 2010). However, this study used artificial avatars and did not involve interaction with live partners.

Other fMRI and lesion studies have identified regions of the brain that are likely to be responsible for building and/or maintaining representations of others′ beliefs and goals, including right-hemisphere structures in temporo-parietal areas such as the posterior temporal sulcus (pSTS) and the TPJ, in addition to certain medial (Saxe and Wexler 2005; Saxe and Powell 2006; Van Overwalle and Baetens 2009, for review) and especially ventromedial (e.g., Gregory et al. 2002; Atique et al. 2011) prefrontal areas. A recent study showed that these same regions may be involved in generating and inferring communicative intentions during live nonlinguistic communication (Noordzij et al. 2009). This is consistent with other recent studies that used relatively realistic social interaction (for review, see Hari and Kujala 2009) and which have shown increased activation in social cognition and reward brain areas (Redcay et al. 2010). In sum, although we have learned much about the various brain systems involved in processing referential ambiguity and in mentalizing, there is still little understanding of when, and how extensively, mentalizing networks might be activated when participating in realistic conversational interaction.

**Figure 1.**
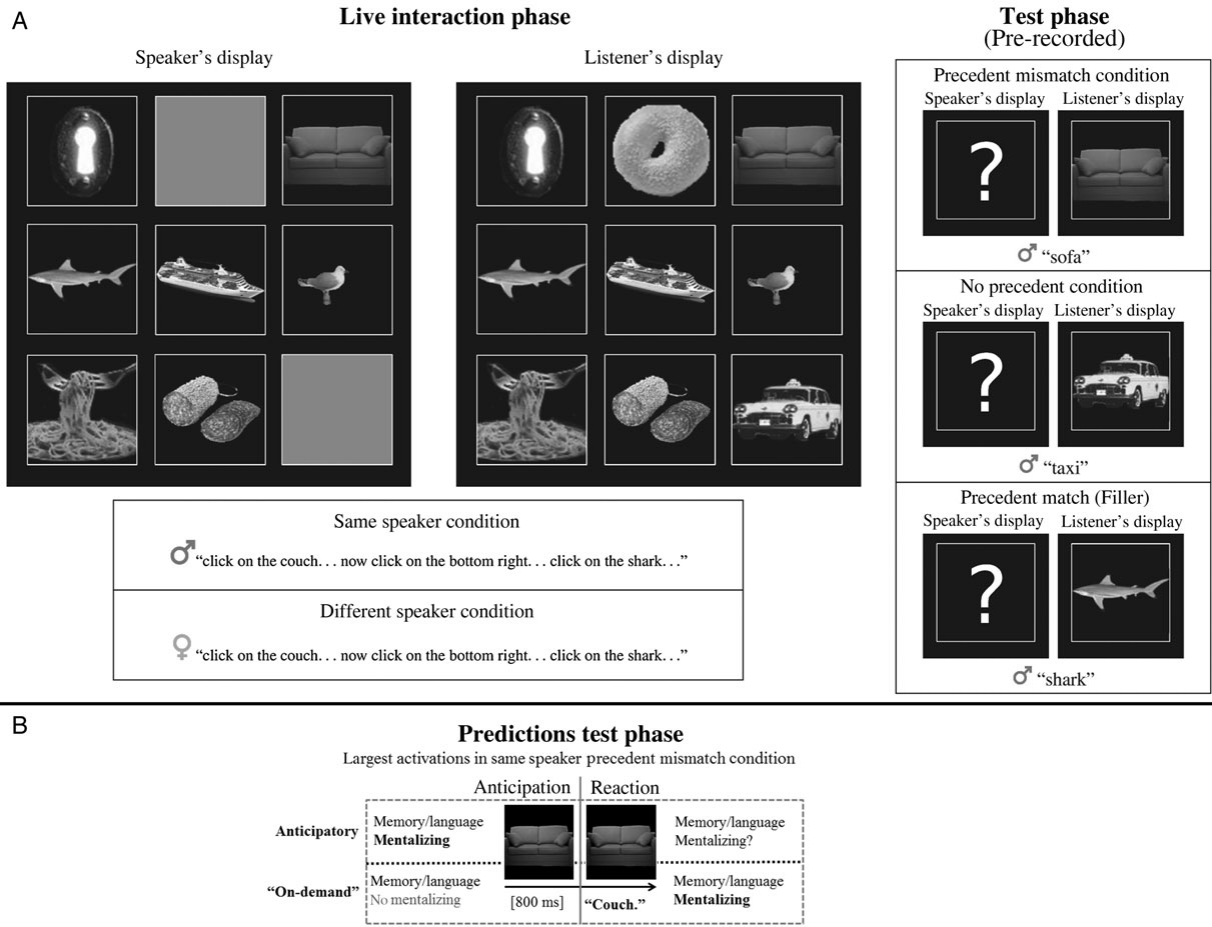
Panel *A*: Design and example displays and speech in the interactive (left) and test (right) phases. Stimuli were presented in color during the experiment. The speaker's view was implied by the speaker's behavior without being seen by the participant and is presented here for clarity. Physical stimuli in the test phase were identical for the 4 experimental conditions (same-/different-speaker precedent match/no precedent) over participants, but test trials were never repeated within a single participant. Panel *B*: Visualization of predictions from the anticipatory and “on-demand” view of mentalizing about which areas are expected to be more active in the same-speaker precedent mismatch than the other conditions during different parts of the test phase.

The absence of neuroimaging studies on conversational language processing reflects the existence of a number of technical and logistical challenges that have imposed a barrier to this kind of research. First, the required signal-to-noise ratio for neuroimaging data analysis typically necessitates a larger number of trials compared with behavioral studies, as well as a high level of control over the stimuli and stimuli presentation timings, in order to reduce any additional sources of variability. This need for large numbers of highly controlled trials is at odds with the characteristics of naturalistic interaction with live conversational partners, where it is difficult to predict what speakers will say and when they will say it. Furthermore, identifying the brain networks involved in the processing of conversational speech requires a neuroimaging technique that provides adequate spatial and temporal resolution. We surmounted these obstacles by using MEG with a novel communication-game paradigm “do-I-see-what-you-mean?” (see Fig. 1, Panel *A*) that enabled spontaneous, quasi-naturalistic conversation with trained confederates, but which still allowed us full control over stimulus characteristics and timing through interleaving prerecorded speech with live speech. Critically, we implemented this interleaving in a way that would lead participants to believe that they were experiencing a live interaction including only spontaneously produced speech by real participants.

The experiment alternated between blocks of trials comprising a "grounding" phase, characterized by spontaneous interaction between the participant and 1 of 2 confederate speakers, followed by a testing phase in which participants heard speech from 1 of the 2 speakers that was (unbeknownst to them) prerecorded and not produced live. During interactive grounding phases (*n* = 42 of interactions in each phase), participants built up temporary referring precedents (e.g., agreements to call a particular object a “couch”; see Brennan and Clark 1996) through live interaction with 1 of the 2 confederate speakers (1 male and 1 female) regarding how images presented on separate screens were to be named (Fig. 1, Panel *A*, left and middle panel). On trials of the subsequent test phase (*n* = 26 of trials in each phase), both the participant and the confederate speaker allegedly saw 1 object on their respective screens (Fig. 1, Panel *A*, right panel). In critical "precedent mismatch" trials, the participant saw a target object from the interactive grounding phase and then heard either the same or the other (confederate) speaker name their own object (prerecorded speech), but using a different (mismatching) term from the one established for the target object during grounding (e.g., using the term “sofa” rather than the established term “couch” to refer to an object; Fig. 1, Panel *A*, right, top panel). Based on the description the speaker chose, the participant had to decide whether or not the speaker was looking at the same object.

In short, we manipulated 2 factors during the critical test-phase trials (in relation to the grounding phase): Firstly, we manipulated whether the same or different (confederate) speaker would name the test pictures (blocked per test phase), and secondly, we manipulated (1) whether the current naming did not match a previously established precedent or (2) whether no precedent had been established at all. That is, in some grounding trials, objects had been referred to by their location (e.g., “top left”), potentially generating interactive memory traces for that object, yet without a naming precedent (see Fig. 1, Panel *A*, right, middle panel). These “no precedent” trials served as baseline conditions for the same- and different-speaker conditions, respectively. Additionally, "precedent match" filler trials (and a number of other catch trials) were added as well to each test phase to ensure that participants would not learn to expect a precedent mismatch on a majority of trials (see Fig. 1, Panel *A*, right, bottom panel; see also Materials for detailed information).

Importantly, a speaker might use a new (mismatching) term for different reasons. The possibility that the speaker might be gazing at a different object provided a cooperative reason why the same speaker, who set up the precedent in the first phase, might use a new term; they might want to indicate that they now see a different picture than before. This could be inferred via mentalizing. In contrast, if a different speaker had established the precedent, the current speaker might simply have different preferences for naming things, so in that case, it is more probable that the speaker is looking at the same picture as the participant. The task made it important for listeners to keep track of who said what, because whether the name they hear is a match or a mismatch (by the same or a different speaker) provides relevant information for deciding whether the speaker sees the same or a different picture. It is important to note that listeners were informed before each phase of test trials whether or not they would hear the same speaker as in the preceding interactive phase or the other speaker. This allowed for consistent person-specific retrieval of previous interactions and possibly anticipation of the speaker's intentions.

Different predictions can be made for the different conditions about the activation time-course of functional neurocognitive networks. It is important to point out, however, that in order to separate the various functional networks and their activation dynamics, we had to rely on the substantial body of previous neuroimaging research about how brain areas relate to function. Thus, the functional separations of episodic memory, language, and mentalizing networks we propose here have to be understood as well-founded, yet hypothetical assertions. Before fleshing out the crucial hypotheses about the time-course of mentalizing, we spell out specific predictions about the time-course of activation of memory and language networks. These predictions are rather independent of the different views regarding the anticipatory vs. “on-demand” role of mentalizing but also relate to speaker-specific common ground vs. more generic speaker-independent processing.

First, the visual stimulus would elicit retrieval of any existing precedents (e.g., couch), if present, before hearing any speech, simply on the basis of the episodic memory of the previous interaction (e.g., Baddeley 2000). Such situated multimodal episodic short- and long-term representations have been associated with medial temporal lobe function (e.g., Squire and Zolamorgan 1991; Olson et al. 2006), allowing viewpoint-dependent retrieval of an object in its visuo-spatial context (e.g., Schmidt et al. 2007; Sulpizio et al. 2013), including the “negotiated” name for the object (e.g., Duff and Brown-Schmidt 2012 for review), along with the identity of the speaker (Imaizumi et al. 1997). Because listeners knew in advance of each test phase which speaker would be speaking, we expected stronger retrieval of the precedent in the same-speaker condition (i.e., speaker identity serving as a further retrieval cue). This could be reflected by differential activation in the middle temporal lobe (e.g., Imaizumi et al. 1997; Olsen et al. 2009) and the temporal poles (Imaizumi et al. 1997), that is, stronger anticipatory episodic retrieval, in conjunction with executive function areas in lateral prefrontal cortex (Sakai and Passingham 2004; Kessler and Kiefer 2005). Importantly, episodic retrieval may lead to anticipation of a specific linguistic expression in the right temporal lobe, which has been proposed in conjunction with right lateral prefrontal cortex (Tourville and Guenther 2011) as a locus for linguistic predictions and integration based on pragmatic (e.g., Gardner et al. 1983; Federmeier 2007) and/or visual episodic context (e.g., Marini et al. 2005). Such activations related to memory retrieval would be elicited by viewing the picture, so they can occur from the moment that the picture is presented but will probably be on-going throughout the trial. Furthermore, such activations would indeed reflect speaker-specific rather than generic speaker-independent memory processing. However, this fulfills a necessary but not a sufficient condition for evidencing the engagement of common ground. As pointed out earlier, it could merely reflect context-specific encoding and retrieval of representations that are shared without being known to be shared. Mentalizing processes, in contrast, would be a more genuine indicator of common ground use.

With regards to hypotheses concerning the involvement of mentalizing, previous research supports a prediction of greater involvement in the condition where the same speaker fails to match an established precedent, as this is the situation in which common ground is apparently violated (Metzing and Brennan 2003; Kronmüller and Barr 2007). In contrast, situations where no precedent had been established or where a different speaker fails to match a precedent would require corrective mentalizing to a much lesser degree (if at all). Thus, observing the strongest mentalizing brain network activation in the same-speaker, precedent mismatch condition would corroborate the general view that participants make use of common ground processing for resolving referential ambiguity in the current paradigm. To distinguish between an anticipatory vs. an “on-demand” view of mentalizing in common ground processing, we need to establish *when* mentalizing is engaged in relation to language processing (see Fig. 1, Panel *B*). Are perspective-taking processes spontaneously engaged prior to an anticipated communicative event, in the service of generating expectations about what a speaker might say and how she might say it? Alternatively, are they mostly engaged "on demand" after the listener suspects communication failure and makes a conscious effort to reestablish common ground? We therefore examined the timing of activation of mentalizing networks, typically associated with the TPJ, precuneus (PC), and the ventromedial prefrontal cortex (vmPFC, e.g., Gregory et al. 2002; Van Overwalle and Baetens 2009; Atique et al. 2011) to understand the extent to which these mentalizing networks are activated prior to naming by the same speaker or, in contrast, only in response to a precedent mismatch produced by the same speaker.

The patterns of activation timing, co-activation, and oscillatory coupling between distinct brain modules provided us with unprecedented detail about processes of human communicative interaction and further allowed us to disentangle a more partner-orientated from a more egocentric conception of interactive communication. However, we would like to re-iterate that the current segregation between functional brain modules is hypothetical (reverse inference), as it relies on previous neuroimaging research for linking brain areas to function. Our results must therefore be regarded as provisional, requiring further testing and confirmation by subsequent research.

## Materials and Methods

### Participants

We obtained MEG data from 16 British participants (8 males), all of whom reported speaking English as their native language. They were recruited from the participant pool of the psychology department of Glasgow University, were paid £6 per hour for their participation, and gave their informed consent. Data from an additional female participant were excluded because she clicked on the wrong picture too often (22 times) in the interactive phase (see Procedure).

### Materials

We gathered 320 pictures (from the Internet) that were given 2 plausible names in an informal pilot as the experimental pictures (see Table 1 for examples). The 2 names for the experimental pictures were selected to be as balanced as possible. The more dominant name was always used the first time the object was named (in the interaction phase, see below). This was done to preclude the explanation that the speaker had thought of a better way to name the picture. We selected 640 other pictures as filler items that were given a name that was clearly dominant in the informal pilot. These pictures were used for 5 different categories of fillers. First, to make sure that not all pictures that were seen in the interactive phase were named differently in the subsequent test phase, we used precedent match fillers (Table 1, third row). We used 80 pictures to appear twice in the interactive phase and once (with the same name) in the following test phase. Second, in order to demonstrate to participants that different pictures could be named in the same way in the 2 different phases, we added the "category filler" condition (20 pairs of pictures, Table 1, fourth row) with 2 different pictures from the same category (e.g., piano and violin), 1 appearing in the interactive phase and 1 in the subsequent test phase, which were both named by their superordinate term (e.g., musical instrument). Furthermore, 100 pictures only appeared in the test phase, 40 of which were named correctly ("new correct fillers," Table 1, fifth row), and the other 60 were named incorrectly ("new incorrect fillers," Table 1, sixth row). The names used in the incorrect filler condition were related to the real names for the pictures (e.g., jar—glass) so that participants needed to pay attention to spot subtle differences. Next to that, 420 pictures were used as "pure fillers" (Table 1, bottom row). These appeared once in the interactive phase but were never named.

**Table 1:** Overview and examples of the experimental and filler items used

Items	Number	Interactive phase	Test phase	Example picture
Experimental precedent mismatch	160	Sofa	Couch	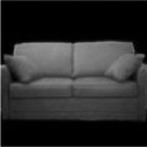
Experimental no precedent	160	Top right (e.g.)	Flame	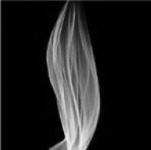
Precedent match fillers	80	Pear	Pear	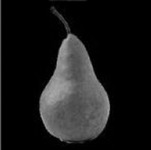
Category fillers (pairs)	20	Musical instrument	*absent*	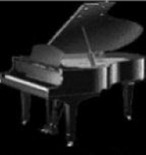
		*absent*	Musical instrument	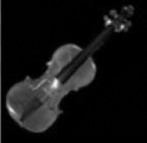
New correct fillers	40	*absent*	Phone	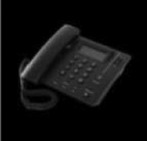
New incorrect fillers	60	*absent*	Glass	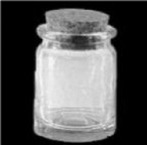
Pure fillers	420	*not named*	*absent*	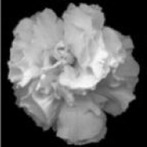

Note: See Materials for descriptions of the types of items. Example pictures (last row) are presented in black and white but were presented to participants in color. The third and fourth columns indicate how the picture was referred to in the respective phases (if the picture was absent or not named in one of the phases, this is indicated in italics). Experimental items are presented in the first 2 rows and the different filler categories in the last 5 rows.

The names for the test phase (see Procedure) were recorded, divided equally between the 2 confederates. Some of the filler (but no experimental) names were recorded with a hesitation, to make participants believe the pictures were named on the spot.

### Procedure

Participants were first prepared for the MEG. HPI coils were attached to the participant's head behind the right and left ears, above the nasion, and on the right- and the left-hand side of the forehead. The coil positions and head shape were digitized before the scan using the Polhemus program and stylus (Polhemus Isotrak, Kaiser Aerospace, Inc.). Digitization is standardly used and allows to determine the head position in the MEG at the start and end of each block (maximum movement tolerated was 0.5 cm) and enables co-registration with the structural MRI for later source localization. Those participants that did not have a structural MRI were scanned after the experiment using a 3T Siemens Trio MRI (Siemens Medical Solutions). Preparation took about 45 min on average, during which the experimenter collected the confederates (instructed fourth-year psychology students, 1 female and 1 male) and introduced them to the participant. Participants were told that these 2 speakers would interact with them via a microphone from separate rooms. After 2 practice blocks, in which participants experienced the role of both listener and speaker, they were presented with 5 20-min runs consisting of 4 blocks each. The order of blocks was randomized per participant. Each block consisted of an interactive phase followed by a test phase. The speaker for each phase was always announced before the start of the phase and remained the same throughout the phase.

In an interactive phase, the speaker/confederate (following a script) asked the participant to click on pictures on the screen using an optical track ball. Participants were told they could interact with the speaker and ask questions. Participants always saw 9 pictures on the screen (see Fig. 1, Panel *A*, middle), and speakers were allegedly seeing the same stimuli and had to name those pictures marked by a red frame (see Fig. 1, Panel *A*, left). The speaker/confederate indicated most pictures by their name, but some by their location (e.g., “bottom right”). In the latter case, pictures in the marked location allegedly were obscured from the speakers view (see, e.g., Fig. 1, Panel *A*, left panel, bottom right picture). In each interactive phase, the speaker/confederate named 13 pictures twice in exactly the same way (later serving as precedent mismatch trials or as precedent match fillers) and 8 pictures twice by their location (later serving as no precedent trials).

In the test phases, the speaker/confederate was the same as for the preceding interactive phase in half of the cases and different in the other half. The participant saw only 1 picture at a time on the screen (see Fig. 1, Panel *A*, right). The speaker/confederate allegedly also saw only a single picture and named that picture (e.g., “sofa”). Participants were asked to indicate whether the speaker's picture was the same as or different from their own, using their dominant hand on the trackball (thumb for same picture and ring finger for different picture). They were not allowed to talk to the speaker. In reality, all utterances of the speaker/confederate in the test phase were recorded beforehand. Each trial started with a fixation cross in the middle of the screen, followed by presentation of the picture at the same location. In experimental trials (precedent mismatch or no precedent), the picture was always presented for 800 ms before the recorded name was played and stayed on the screen until a response was given. For filler trials, the preview interval before presentation of the name was varied. Each test phase started with 2 filler trials, followed by a random presentation of 8 precedent mismatch trials (named differently than in the interactive phase), 8 no precedent trials (indicated by their location in the interactive phase), 4 precedent match fillers (named the same as in the interactive phase), and 4 other fillers (see Materials and Table 1). Note that the physical stimuli in the test phase were identical for the 4 experimental conditions over participants, but items were not repeated within a participant. The different conditions were created by changing the speaker and the particular reference to this picture in the preceding interactive phase. Each experimental condition (same-speaker precedent mismatch; same-speaker no precedent; different-speaker precedent mismatch; different-speaker no precedent) occurred 80 times throughout the experiment.

### Apparatus

MEG data were acquired using a 248-channel (or SQUIDs; superconducting quantum interference devices) whole head magnetometer (4D-Neuroimaging Magnes 3600 WH system) at the CCNi at the University of Glasgow, sampled at 508.63 Hz and band-pass filtered between 0.1 and 200 Hz. Trigger pulses via the parallel port were used to synchronize MEG data acquisition with experimental events. A Panasonic 3-chip DLP projector (PT-D7700E-K) was employed for visual stimulus presentation. Resolution was 1024 × 768 pixels covering a visual angle of 24° horizontal by 18° vertical. Each picture in the 3 × 3 matrices, employed during the interactive phases, covered a visual angle of 3.5° × 3.5°, whereas the single pictures presented during the testing phases covered 4.7° × 4.7°.

### Ethical Statement

All procedures (including consent and participant debriefing) were reviewed and approved by the College Ethics Committee of the University of Glasgow and were in full agreement with APA and BPS guidelines, as well as with the Declaration of Helsinki.

### Data Analysis

Preprocessing and statistical analysis was conducted using the Fieldtrip Matlab^®^ toolbox (Oostenveld et al. 2011) and was in agreement with recently published guidelines for MEG research (Gross et al. 2012). First, epochs were extracted from the MEG for all test phase trials from 500 ms before the picture was shown on the screen (i.e., 1300 ms before sound onset) until 500 ms after the response. Subsequently, linear trends were removed and all epochs were denoised to remove signals generated by the HPI coils. Trials with very large (movement and/or eye) artifacts were removed before PCA/ICA since this procedure can be unreliable if the data contain much noise. On average, only 1 or 2 trials were removed per experimental condition at this stage (maximally 5, no significant differences between conditions). Then, PCA was used to reduce data dimensionality for each participant to 40, 60, or 100 components, which were then subjected to ICA (Oostenveld et al. 2011; Gross et al. 2012). A higher number of components were used if signal and noise could not be separated clearly using the lower number of components. These components were inspected visually and removed if they contained only noise and/or artifacts (e.g., caused by heart beats or eye movements). The average proportion of removed components was 0.20 (range: 0.05 to 0.37). The remainder of the components was used to recreate the MEG signal. After that, individual trials with remaining artifacts were removed manually once more. This resulted in an average of 76.1 remaining trials (range: 69–80 out of 80). The numbers of remaining trials did not differ between the 4 conditions within the same analysis (Fs > 1). To identify the same-speaker "deliberation trials" (see ERF results below), we took all trials from the same-speaker, precedent mismatch condition in which participants gave a "different picture" response, plus the one-third "same picture" responses with the longest RTs per participant. This led to an average of 42.6 deliberation trials per participant (range: 32–66). Finally, 5 or 6 bad channels were interpolated for each participant based on the signal of neighboring channels. These preprocessed data entered the ERF and time-frequency (power) analyses. For coherence analysis, we used preprocessed data without removing components via the standard PCA/ICA approach, since it has been suggested that removing ICs, when preceded by a PCA, distorts the oscillatory phase of the signal (e.g., Castellanos and Makarov 2006). All reported coherence effects are relative between conditions and unlikely to be biased by artifacts (e.g., heart, muscles), assuming a random distribution of artifacts across trials and conditions.

For evoked responses (ERF), trials of the same condition were averaged per participant. These averages were adjusted in relation to a baseline interval of 200 ms immediately prior to picture onset and filtered with a band-pass filter between 0.5 and 35 Hz. For time-frequency representations, the power of each frequency between 2 and 30 Hz (with steps of 1 Hz) was calculated on individual trials over time using a Hanning taper (Grandke 1983) with a window of 4 cycles (changing in length per frequency). For both ERF and time-frequency averages, planar gradient representations were calculated prior to sensor-level analysis. Often it is helpful to interpret MEG fields measured by magnetometers (and axial gradiometers, e.g., Gross et al. 2012) after transforming the data to a planar gradient configuration, that is, by computing the gradient tangential to the scalp. One advantage of the planar gradient transformation is that the signal amplitude typically is largest directly above a source. This transformation is particularly helpful for sensor-level analysis as it also allows for more communality across participants with the same source locations yet with differing orientations (planar gradient represents the focus above the location of the source and not the more orientation-dependent fields around the source). However, for source level analysis, the original magnetometer representation and according lead fields were employed. To test for statistically significant differences between conditions and reduce the multiple-comparison problem, we used the cluster-based approach implemented in the Fieldtrip toolbox (Maris and Oostenveld 2007). This robust method reduces the multiple-comparisons problem and controls family-wise error across subjects in time and space. To examine differences between experimental conditions, paired *t*-tests are performed for each time-point, channel, and frequency (for time-frequency analyses) with a threshold of *P* < 0.05. Significant clusters in time, space, and frequency are identified on the basis of proximity (neighbors) in all dimensions of the cluster. Cluster statistics are calculated by taking the sum of *t*-values in every cluster. To obtain a *P*-value for each cluster, a Monte Carlo method is used to evaluate how extreme the cluster statistics of the 2 conditions are compared with random partitions of the samples. The proportion of random partitions that results in larger cluster statistics than the observed one is the *P*-value. The threshold was fixed to *P* = 0.05.

We employed 2-step analyses for emulating the interaction between 2 factors in time and frequency analyses. We first calculated a *t*-statistic for the difference between 2 conditions, for example, precedent mismatch vs. no precedent trials for each participant separately and then included the outcomes (*t*-values) of this first step statistic into a group statistic that compared a second difference, for example, same vs. different speaker (note, the first level *t*-statistic was calculated separately for the same- and different-speaker condition at the individual level). The comparison at the group level followed the robust statistics approach described earlier. The described 2-step analysis approached the interaction between speaker (same/different) and precedent (precedent mismatch/no precedent).

To identify sources underlying the sensor-level effects, individual single-shell (Nolte 2003) head models were generated based on the individual MRI aligned with the MEG sensor array via the conducted head digitization. Voxel size was 6 mm, and all individual head models were normalized to a standard brain prior to analysis. ERF sources were identified using a Linearly Constrained Minimum Variance (LCMV) beam former (Van Veen and Buckley 1988), where we calculated a common LCMV filter for all 4 conditions (to increase SNR) per participant. This common filter was then used to transform (“beam”) the individual conditions into source (voxel) space for comparisons between conditions. For identifying generators of theta oscillations, we employed Dynamic Imaging of Coherent Sources (DICS) beam formers (Gross et al. 2001). In this case, we were able to use condition-specific spatial filters that could potentially reveal qualitative differences between conditions. DICS was also employed for localizing (inter-trial) phase-coherent sources in theta (4–6 Hz) by means of cross-spectral density matrices in relation to particular reference signals (see Gross et al. 2004; Kessler et al. 2006). For statistical testing of source-localizations underlying ERF and time-frequency effects and coherent sources, we used the same cluster-based approach, in this case only clustering over voxels. Time windows and frequency ranges (in case of time-frequency sources) were chosen based on significant sensor-level effects. Sources identified in theta-power analysis were employed as references for theta coherence analysis. For this type of analysis, we reduced the multiple-comparison problem by using a "false discovery rate" (FDR) approach, since it has been suggested to be more sensitive to spatially localized effects compared with a bias toward more widespread effects in cluster-randomization (Groppe et al. 2011).

## Results and Discussion

**Figure 2.**
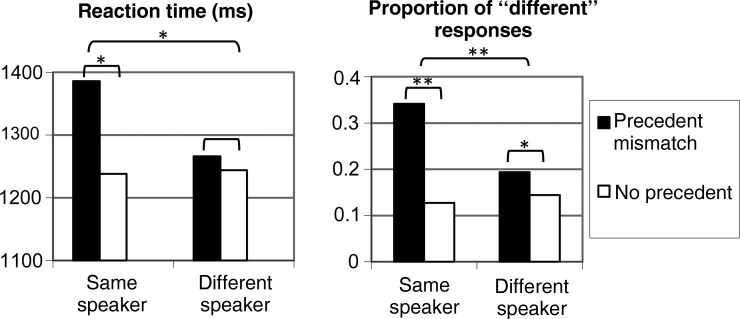
Behavioral responses; RT (left) and choice responses (“different” or “same” picture; right). *P < 0.05; **P < 0.001.

Confirming the results of previous studies (Metzing and Brennan 2003; Kronmüller and Barr 2007; Matthews et al. 2010), our behavioral results indicated that listeners experienced greater confusion for precedent mismatches produced by the same speaker as compared with those produced by a different speaker. Figure 2 shows that both reaction times and choices revealed an interaction between speaker (same/different) and precedent (mismatch/no). ANOVAs confirmed the prediction of a larger precedent mismatch vs. no precedent effect in the same speaker than the different-speaker case for RT (longer RTs, *F*_1,15_ = 8.43, *P* = 0.011) and for response choices (more "different" responses; *F*_1,15_ = 21.15, *P* < 0.001). These interaction effects allowed us to specifically search for the neural substrates involved in generating these effects. We primarily analyzed MEG signals in the frequency domain since this type of analysis (in contrast to averaging, i.e., ERFs) is sensitive to evoked as well as to induced brain signals (e.g., Pfurtscheller and Lopes da Silva 1999). We found strong effects in the theta band (4–6 Hz) that reflected widespread differences in cortical activity across conditions. These will be reported in the next section, whereas the more confined effects in alpha (9–13 Hz) and gamma (66–78 Hz) are reported in Supplementary Figure S1.

### Theta Oscillatory Effects

#### Theta Power (Sensor and Source Space)

**Figure 3.**
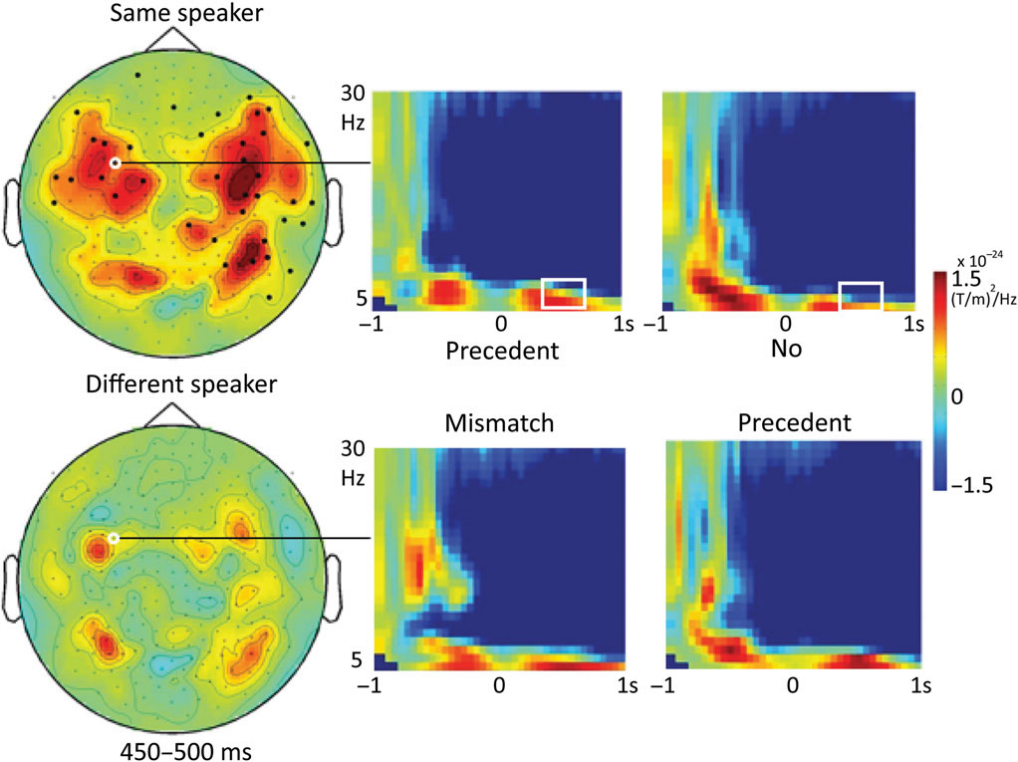
Time-frequency representations. Effects were found in theta (4–6 Hz) between 350 and 650 ms, only for the precedent effect within the same-speaker condition (top row), but not within the different-speaker condition (bottom row). Colors in the topographical plots on the left indicate differences in power (precedent mismatch minus no precedent), relative to a baseline time window (−1000 to −800 ms, just before display of the picture). Channels participating in the significant cluster in a representative time window (450–500 ms) are indicated by black dots. White circles indicate the channel shown in the 4 time-frequency plots on the right, showing power, relative to the baseline time window for each of the 4 conditions. White squares indicate the location of the effect in time and frequencies. Results for alpha and gamma frequencies are reported in Supplementary Figure S1.

Analyses were time-aligned to the onset of the spoken expression, such that negative values of the time variable represent processes taking place during the picture preview (pre-naming), whereas positive values represent processes taking place after onset of the verbal expression (post-naming). Consistent with other studies reporting theta oscillations in the context of episodic working memory and language processing (e.g., Bastiaansen et al. 2002; Hagoort et al. 2004; Bastiaansen et al. 2005; Jensen and Colgin 2007; Fuentemilla et al. 2010; Giraud and Poeppel 2012), we found significant modulations of frequencies between 4 and 6 Hz. A time-frequency analysis at the sensor level between −800 and 1000 ms in the range of 2–30 Hz revealed a significant cluster (*P* = 0.012) in the theta range (4–6 Hz) for the precedent mismatch vs. no precedent comparison within the same-speaker condition, in a time window around 350–650 ms after naming onset (Fig. 3, top row). The corresponding comparison for different speaker did not reveal a significant effect in theta (Fig. 3, bottom row) or any other frequency (see Supplementary Fig. S1), corroborating the special status of the same-speaker, precedent mismatch trials observed in the behavioral data.

**Figure 4.**
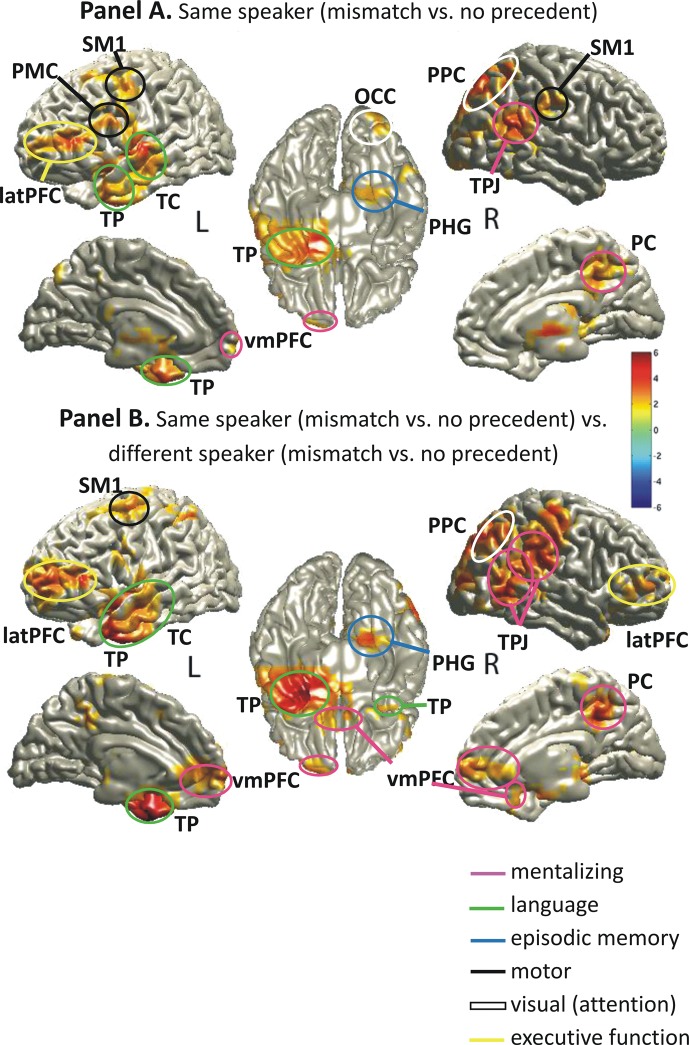
Theta-power sources localized for the post-naming interval by means of DICS (see Methods). Sources in red show a power increase in 3–7 Hz for the same-speaker precedent mismatch as compared with the no precedent condition (Panel *A*) or as a result of an interaction between speaker and precedent (Panel *B*). The color-coded scale represents *t*-values. Labels are L for left and R for right hemisphere; SM1, primary sensori-motor cortex; PMC, premotor cortex; PPC, posterior parietal cortex; OCC, occipital cortex; latPFC, lateral prefrontal cortex; TP, temporal pole; TC, temporal cortex; TPJ, temporo-parietal junction; PHG, parahippocampal gyrus; PC, precuneus; vmPFC, ventromedial prefrontal cortex. Further explanations are given in the text and Supplementary Table S1.

We localized the sources of the observed sensor-level theta effect (same speaker: precedent mismatch vs. no precedent; see Fig. 3, top row) using DICS (see Methods) collapsing over a post-naming time window between 200 and 800 ms (to include ∼3 theta cycles) and 3 to 7 Hz. We chose these parameters to cover the maximum of the sensor-level effects across time samples and frequencies. The findings for the same-speaker, precedent effect (1 spatially distributed cluster, *P* = 0.028) are shown in Figure 4, Panel *A* (and Supplementary Table S1, Panel *A*) and revealed sources in areas that previous research (see Introduction) has related to: (1) mentalizing (right TPJ, ventromedial prefrontal cortex vmPFC, right PC), (2) episodic working memory including executive function (right parahippocampal gyrus PHG, left lateral (lat)PFC), (3) language (left temporal cortex TC, including left temporal pole TP), (4) attention (right posterior parietal cortex, PPC), and (5) motor functions (left lateral premotor and motor cortex, PMC). This source pattern conformed very strongly to our expectations regarding functional processing networks interacting in the post-naming interval (see Introduction).

In order to further strengthen our pattern of results, we conducted a two-step analysis (see Methods) with the frequency characteristics and within the time interval described earlier in order to analyze the speaker-by-precedent interaction in source space. This analysis revealed significant interactions between speaker and precedent that showed a strikingly similar pattern (1 spatially distributed cluster, *P* = 0.008; Fig. 4, Panel *B*; Supplementary Table S1, Panel *B*) to that of the simple contrast in the same-speaker condition described earlier. Additional effects in the two-level analysis extended to the right lateral PFC, typically associated with executive functions in working memory. Effects also seemed to be more pronounced and extensive in core areas around the right TPJ, PC, and vmPFC, previously linked to mentalizing (see Introduction). Episodic working memory in right PHG and the left latPFC also revealed more pronounced levels of significance in this two-step analysis. In the left language areas, the focus of significance was shifted toward TP but still comprised middle TC.

Overall, the pattern across the 2 types of analysis is reassuringly consistent and highlights the involvement of typical mentalizing, episodic working memory, and language areas (conforming to previous research, see Introduction); a pattern that is highly specific to the same-speaker, precedent mismatch condition and to the post-naming interval. Note that observing the same basic pattern of theta-power effects in the interaction as well as in the simple contrast rules out the possibility that a negative effect for the different-speaker conditions may have manifested as an overall positive effect compared with the same-speaker conditions, thus, potentially driving the interaction effect. This is further in line with the reported sensor-level effects (see Fig. 3), where the same-speaker, precedent mismatch condition revealed the strongest theta-power increases.

**Figure 5.**
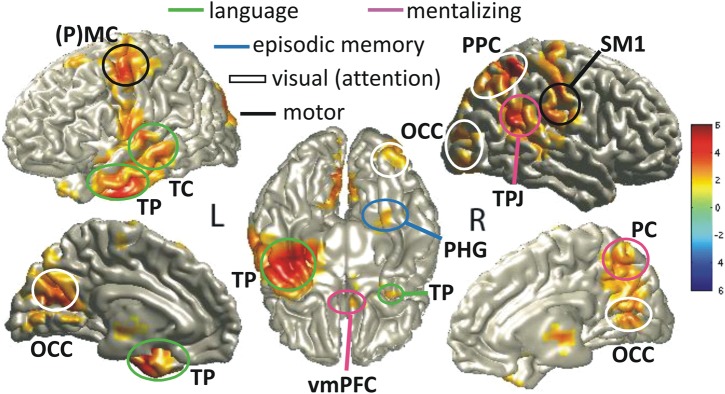
Theta-power sources before and after naming, localized by means of DICS (see Methods). Sources in red show a power increase in 3–7 Hz for contrasting same-speaker, precedent mismatch vs. same-speaker, no precedent in the post-naming interval compared with the same contrast in the pre-naming interval. Within the same-speaker condition, we compared precedent mismatch vs. no precedent conditions separately for the post- and the pre-naming intervals and for each participant (first step) and then employed a group-level statistic (second step) for comparing the 2 intervals. The color-coded scale represents *t*-values. For source labels, see Figure 4. Further source details are reported in Supplementary Table S2.

Finally, in order to substantiate whether the sources observed for theta power in the post-naming interval, particularly in mentalizing areas, were indeed significantly less active in the pre-naming interval, we compared the 2 time periods directly by means of another two-step analysis. For the pre-naming interval, we chose a time window between −800 and −200 ms, of the same length as the 200- to 800-ms post-naming interval. The first step comprised of comparing the precedent mismatch to the no precedent condition for same speaker (controlling for low-level sensory differences) both pre- and post-naming. The pre-naming contrast was compared with the post-naming contrast in the second step. This led to 1 positive cluster (*P* = 0.006), see Figure 5 (and Supplementary Table S2). The results further corroborate our interpretation that most theta sources observed in the previous analysis and particularly those in typical mentalizing (and related social) areas such as TPJ, PC, TP, and parts of the vmPFC showed significantly stronger activation for same-speaker, precedent mismatch vs. no precedent trials in the post-naming interval. The right PHG and bilateral visual areas (occipital OCC) also revealed significantly stronger theta power for this comparison in the post-naming interval. Based on the existing literature (see Introduction), this suggests stronger episodic retrieval in the right hemisphere along with stronger visual processing in response to the naming mismatch. Left TC activation could indicate that a mismatch with an anticipated precedent (as compared with just hearing a new name without any precedent) may have led to more prominent language area activation than building up anticipation for a certain term pre-naming. As a form of "reality check," left motor areas (MC) also showed up in this analysis. This was expected because no difference in motor response should be present pre-naming, whereas after naming, response processing was stronger for precedent mismatch than for no precedent trials.

#### Theta Phase-Coherence (Source-Space)

**Figure 6.**
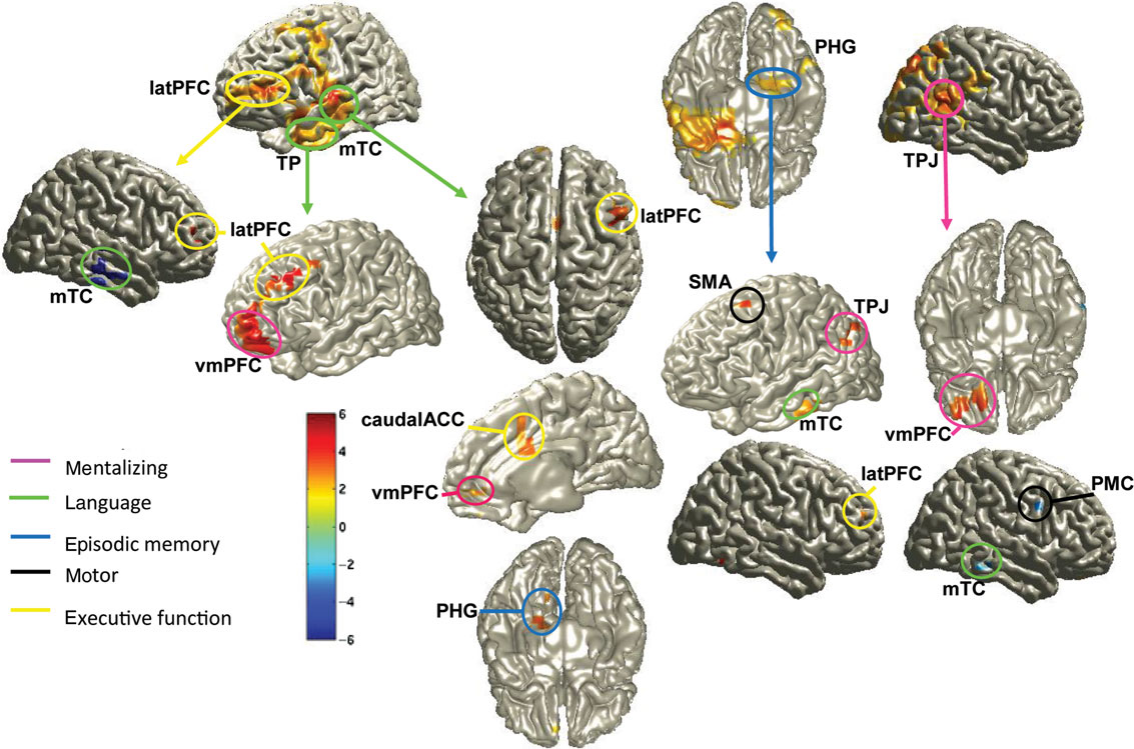
Theta phase-coherence (4–6 Hz) localized for the post-naming interval by means of DICS (see Methods). Differences in coherent sources between same-speaker, precedent mismatch minus same-speaker, no precedent (FDR-corrected significant *t*-values) in reference to theta-power sources in latPFC, left TP, left mTC, right PHG, and right TPJ as identified in the reported power analyses (see Fig. 4). The color-coded scale represents *t*-values. New source labels in this figure are ACC, anterior cingulate cortex; SMA, supplementary motor area. For all other source labels, see Figure 4. Red-yellow sources denote stronger coherence in the same-speaker, precedent mismatch compared with the same-speaker, no precedent condition, whereas blue sources denote the opposite effect. Further explanations in the text and Supplementary Table S3.

To obtain a picture of the functional connectivity between these various brain areas, we analyzed phase-coherence in the 4–6 Hz band between 200 and 800 ms post-naming (see Methods). We contrasted precedent mismatch and no precedent conditions for the same speaker to identify cortical areas that revealed coherence differences relative to a particular reference site. The overall pattern of coherence is shown in detail in Figure 6 (and Supplementary Table S3) and reveals functional connectivity effects (statistical maps of significant theta phase-coherence effects) in relation to “seed” areas (reference sites) taken from the previous theta-power source analyses. Red color denotes areas that are coupled significantly stronger with the respective seed area in the mismatch compared with the no-precedent condition (mismatch > no precedent), whereas blue color denotes the reverse effect (mismatch < no precedent).

Left TP and left TC were coupled to left PHG and left/right latPFC, and to vmPFC, which we interpret based on the existing literature (see Introduction) as the functional coupling between subnetworks related to language, episodic working memory, and mentalizing. This functional interpretation is further corroborated by the statistically significant coupling between right PHG and areas in latPFC, mTC, and left TPJ. Caudal anterior cingulate cortex has been related to conflict monitoring, and the currently observed coupling with temporal cortex could reflect conflict resolution between current and retrieved naming.

Importantly, when using the right TPJ as a reference site, we found the left vmPFC cortex to be significantly more coherent (mismatch > no precedent). This corroborates the notion that information exchange within the mentalizing network was engaged more strongly for precedent mismatch trials by the same speaker compared with when no precedent had been established during the interaction. In addition, and somewhat surprisingly, significantly less coherence (mismatch < no precedent) was found in relation to right middle TC and to right PMC. Reduced theta phase-coherence in mismatch compared with the no precedent condition could suggest active decoupling between 2 areas and could reflect suppression (e.g., Gross et al. 2004). The present result could be interpreted as TPJ suppressing predictions that were generated by the right hemisphere (particularly TC) during the pre-naming interval (e.g., Gardner et al. 1983; Marini et al. 2005; Federmeier 2007 for review; Tourville and Guenther 2011). While this remains speculative because coherence is a correlative measure that does not allow inferring a direction of influence, the right middle TC also reveals a similar pattern of decoupling in relation to the left lateral PFC (Fig. 6), supporting the notion of top-down suppression of a “wrong” linguistic prediction after the same speaker used a term that mismatched with the expected precedent.

### Analysis of “Same-Speaker Deliberation” Trials

So far, the results reported for the oscillatory domain have revealed stronger theta activity in the post-naming interval for same-speaker, precedent mismatch trials as compared with no precedent trials, but no significant differences at all were observed in the pre-naming interval and a direct (theta power) comparison showed that the post-naming effects were significantly stronger compared with pre-naming. Importantly, the post-naming effects in theta-power and -coherence have included areas and their coupling, which previous research has identified as core mentalizing areas (see Introduction). The results so far, therefore, support the notion of “on-demand” involvement of perspective taking and mentalizing in language processing.

However, it might be difficult for slow theta effects to reach significance in the relatively short (800 ms) pre-naming interval. Furthermore, to the extent that mentalizing might be taking place equally across all conditions, specifically in the pre-naming interval, these networks might not show up in any cross-condition comparisons. Although this seems unlikely, given the robust mentalizing differences observed in the post-naming interval, we made a final attempt to identify anticipatory brain activity in general and activity that could be related to mentalizing in particular. To create the best opportunity for finding mentalizing effects, we specifically targeted those trials within the same-speaker, precedent mismatch condition in which the behavioral evidence suggested engagement of mentalizing processes, either through a “different picture” response or through a “same picture” response with a particularly slow response time (see Methods for details). As these are trials where some sort of deliberation has evidently occurred, we refer to these particular trials as "same-speaker deliberation trials." We compared these trials again to the same-speaker, no precedent trials, yet by selecting this subset with the highest probability of deliberation, conform to our behavioral indicators, any activation of mentalizing networks in the pre-naming interval that may have occurred should now become apparent.

**Figure 7.**
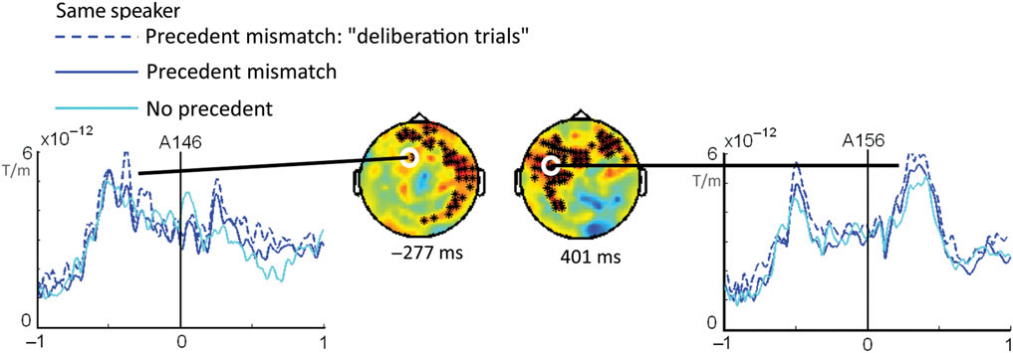
Sensor-level ERF effects. Sensor-level ERF comparisons for same-speaker deliberation trials (dotted dark blue line) compared with the same-speaker, no precedent trials (light blue line). Black asterisks indicate channels participating in significant clusters for this contrast in a representative time window. Two different time windows are shown: left = pre-naming; right = post-naming. The solid dark blue line representing all same-speaker, precedent mismatch trials is added for comparison. See also Supplementary Figure S2 for all sensor-level ERF results.

Note that pre-selecting these trials is especially favorable toward a central, anticipatory view of mentalizing and works against more “on-demand,” egocentric accounts. If participants give a "different picture" response, they show evidence that they took the perspective of the speaker and noted that she probably sees a different picture if using a different name than before. Slow responses at least indicate that participants probably did not expect this name, possibly because they used mentalizing to anticipate a certain name. Hence, if even this subset of trials does not support anticipatory mentalizing activity in the pre-naming interval, this would provide a strong argument against the idea that listeners use mentalizing spontaneously to generate speaker-specific linguistic predictions. Deliberative trial selection was applied separately for time- and frequency-data, yet only evoked responses in the time-domain (ERF; cf. ERP) revealed significant anticipatory effects in the pre-naming interval (Fig. 7). Complete ERF results are reported in Supplementary Figure S2.

#### ERF Analysis of Same-Speaker Deliberation Trials (Sensor and Source Space)

**Figure 8.**
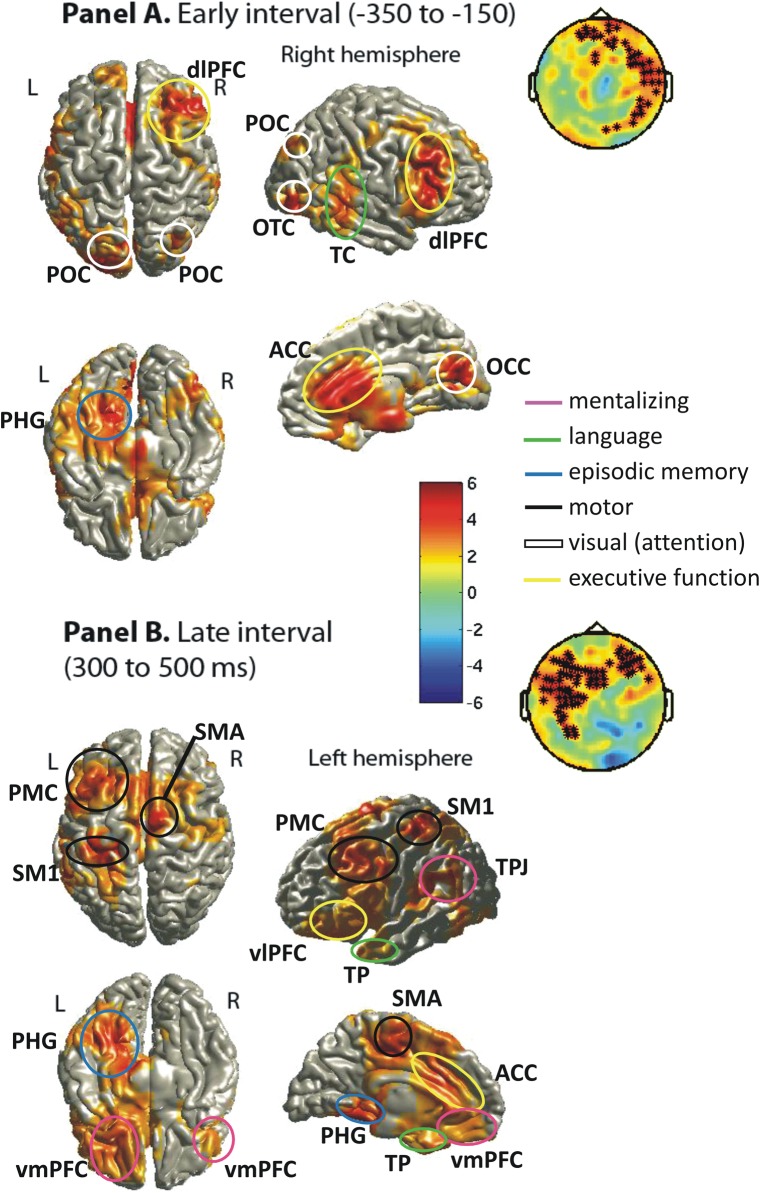
Significant sources for deliberation trials vs. no precedent (same speaker) using LCMV beam formers (see Methods). Panel *A* shows sources for the pre-naming interval (together with the corresponding ERF topography from Fig. 7, left). Panel *B* shows the sources for the late interval (ERF topography from Fig. 7, right). The color-coded scale represents *t*-values. Source labels do not conform to Figures 4 and 6 apart from POC, parieto-occipital cortex; OTC, occipito-temporal cortex; vlPFC, ventro-lateral prefrontal cortex. Further explanations are in the text and in Supplementary Table S4.

Focusing on same-speaker deliberation trials, as compared with the same-speaker, no-precedent trials, we found 3 significant clusters between −800 and 1000 ms. Suggesting anticipatory processing, 2 were in the pre-naming preview phase; 1 between −550 and −23 ms (*P* = 0.004), 1 between −306 and 0 ms (*P* = 0.004) and both with a predominantly right-hemisphere topography (Fig. 7, left; Fig. 8, Panel *A*). One cluster (*P* < 0.00001) lasted between 67 and 680 ms after naming, with a maximum amount of significant channels around 400 ms and a predominantly left-hemisphere topography (Fig. 7, right; Fig. 8, Panel *B*). Source analysis was then employed for both the pre- and post-naming interval to examine whether the anticipatory clusters involved mentalizing in addition to episodic retrieval.

In the early interval, a time window between −350 and −150 ms (before naming onset) was centered on the peak of the sensor-level effect, and 1 significant, spatially distributed cluster was found (*P* < 0.00001). For LCMV beam former analysis of the post-naming interval, a time window between 300 and 500 ms was centered on the peak of the effect at sensor level and another significant, spatially distributed cluster was found (*P* < 0.00001). Conforming to the topography shown in Figure 7 (left), sources for the pre-naming interval were located predominantly in the right hemisphere (see Fig. 8, Panel *A*, and Supplementary Table S4, Panel *A*). Sources comprised areas typically associated (see Introduction) with episodic working memory (right dlPFC, ACC, left PHG), language (right mTC), and visual processing (left occipital temporal cortex OTC, left parieto-occipital cortex POC, right OCC). In contrast, no clear mentalizing activation could be identified according to the typical areas reported in the literature and reviewed in Introduction. This pattern of results is in agreement with egocentric processing, rather than partner-oriented anticipation (see Introduction). PHG involvement in particular suggests that participants retrieved the episodic context associated with the specific target object they were viewing. Differences in visual areas further support the notion that episodic retrieval of previously named objects was more visually detailed. PHG might therefore play an anticipatory role in conjunction with dlPFC for retrieving the episodic context of the interaction with the target object, including information about the speaker and the used name—prior to the current naming of the object (e.g., Imaizumi et al. 1997; Epstein and Kanwisher 1998; Olson et al. 2006; Rankin et al. 2009). The latter is in agreement with the observed effects in typical language-related areas of the middle TC in the right hemisphere, possibly indicating anticipation of the name previously associated with the picture during interaction (e.g., Gardner et al. 1983; Marini et al. 2005; Federmeier 2007; Tourville and Guenther 2011). Effects in ACC are compatible with the notion of anticipation of cognitive effort or conflict (Sohn et al. 2007; Aarts et al. 2008). Due to the substantial amount of precedent mismatch trials (see Table 1), participants may have learned to anticipate conflict with the linguistic predictions they generated based on their previous interaction.

While we found evidence that speaker-specific predictions were generated based on episodic retrieval, it is doubtful whether this activation reflects common ground processing or merely context-specific retrieval (see Introduction). Given the lack of evidence for a difference in activation of TPJ and other mentalizing areas reported in the current literature (see Introduction) between same-speaker deliberation and no precedent trials in this pre-naming period, we must conclude that the overall picture of results is consistent with the general idea that communicatively relevant, partner-specific information can be activated through basic memory processes without mediation by access to common ground in terms of active co-representation of the other's mental states (Horton and Gerrig 2005; Barr et al. 2014).

Sources for the post-naming interval were predominantly left lateralized (Fig. 8, Panel *B*, and Supplementary Table S4, Panel *B*) in concordance with the sensor topography of the ERF effects (Fig. 7, right). Effects in typical motor/premotor areas (PMC) might reflect more intense or more conflicting motor preparation that would fit with ACC involvement, which has been linked to conflict monitoring as well as anticipation (Sohn et al. 2007). PHG also seemed to be involved in both intervals (and across all types of analysis), possibly suggesting that episodic retrieval efforts may have been continuously engaged more strongly in deliberation trials. This is of particular interest in the context of mentalizing effects in left TPJ and vmPFC only “after” naming. Samson et al. (2004) pointed out the particular relevance of the left TPJ for reasoning about others' beliefs, which in the present context only seemed to be engaged “on-demand,” when mentalizing was required to resolve a conflict.

#### Relating ERF and Theta Results

The pattern observed in the ERF analysis of same-speaker deliberation trials complements and corroborates our results based on theta power and phase-coherence. So far, a few of the sources had only been reported in theta phase-coherence analysis. These have now been confirmed in the ERF analysis of deliberation trials: left PHG, left TPJ, caudal ACC, right PMC, and right middle TC (see Figs 6 and 8). Importantly, however, potential mentalizing in the left TPJ was only confirmed for the post-naming interval (see ERF and coherence analyses). Furthermore, an area in the right middle TC that was most likely associated with linguistic predictions in the pre-naming interval (ERF analysis) revealed de-coupling of theta phase during the post-naming interval in relation to left lateral PFC and right TPJ (Fig. 6). This corroborates the proposed notion (see Section on theta phase-coherence) that “wrong” linguistic predictions might be suppressed when common ground is reestablished, that is, when an apparent precedent violation is resolved via mentalizing.

Although our results consistently show activation of areas previously related to mentalizing processes in the post-naming, but not the pre-naming interval, 2 potential concerns could be raised. First, areas such as the TPJ, vmPFC, and precuneus have reliably been related to mentalizing activities but could possibly also reflect other functions as well, since many brain areas participate in more than one cognitive activity. Next to mentalizing, the TPJ, for example, has been related to reorienting of attention to an unexpected stimulus (e.g., Corbetta and Shulman 2002; Mars et al. 2012). In the context of the present paradigm, a mismatching name might be considered an unexpected stimulus, because participants anticipate the previously mentioned name based on memory retrieval of the picture and its context. Insofar as the expectation for a certain name is stronger in the same-speaker condition (because of a tighter contextual similarity), the unexpectedness of the mismatch might be most prominent in the same-speaker, mismatch condition and therefore lead to the strongest TPJ response in that condition. It is difficult to rule out such an alternative explanation directly. However, we found both right and left TPJ to be involved across different analyses. Also, our theta phase-coherence results revealed a coupling between the right TPJ and the left vmPFC, 2 areas that have been strongly associated with the mentalizing network and their direct functional coupling is harder to reconcile with a stimulus expectancy account than with a mentalizing account (see also Mars et al. 2012). Nevertheless, further converging research, using similar paradigms, will be necessary to completely rule out alternative explanations and support our interpretation that more extensive mentalizing is required in response to experimental conditions such as the current same-speaker, mismatch condition. For example, an fMRI study, because of its better spatial resolution, could pinpoint more specifically the part of the TPJ involved, allowing for more precise functional interpretations (see, e.g., Mars et al. 2012). Alas, such an approach would not be able to corroborate our findings in terms of “when” mentalizing is engaged.

A further caveat concerns the fact that no differences in mentalizing areas were found in the pre-naming interval. This is in some aspects a null result, which should be interpreted with care and needs to be replicated by future studies. However, our direct comparison of theta power between the pre- and post-naming interval revealed that most effects (including TPJ) reported for the post-naming interval were significantly stronger compared with pre-naming, which corroborates our current interpretation. Finally, in our ERF analysis of deliberation trials, an extensive network of areas (typically related to episodic memory and language in previous research) was found to be activated in the pre-naming period, showing that our analysis was sensitive and powerful enough to pick up on differences between the conditions in that interval. Still, no typical mentalizing area such as TPJ was involved in this pattern, further corroborating our conclusion that these areas do not appear to be involved in anticipatory processing.

### General Discussion

Our results indicate that brain areas typically related to language, vision, episodic working memory, and mentalizing in previous research are dynamically and jointly involved in encountering and resolving conflict after encountering a reference that mismatches with a previously negotiated precedent by the same speaker. The reported effects on behavior, evoked responses, oscillations, and sources were most pronounced when the mismatch occurred with respect to a precedent established by the same speaker, compared with processing of trials without a precedent or when the precedent had been established by a different speaker. It is important to note that the paradigm employed here was special in making the speaker's identity known to the listener before each block of test trials, thus giving ample opportunity for listeners to mentalize and access common ground to enhance speaker-specific linguistic predictions.

The dynamics of theta oscillations, sources, phase couplings, and evoked responses are fundamentally novel findings in themselves and overall have consistently revealed that listeners do access common ground previously established with a specific speaker (but not another speaker) and that these processes seem to involve speaker-specific episodic retrieval as well as mentalizing. These findings are compatible with most behavioral experiments showing partner-specific processing of referential precedents. However, the results across several, very different analysis approaches in source space (theta-power, -coherence, ERF) also shed light on the mechanisms that are engaged in anticipation of an upcoming linguistic reference in contrast to those mechanisms that are engaged in reaction to an apparent pragmatic violation of previously established common ground. Overall, we found more robust mentalizing effects (evoked and oscillatory theta activity) in the post-naming interval, suggesting that on a majority of trials, participants engaged in enhanced episodic retrieval and, most importantly, mentalizing in response to a perceived violation.

Only when focusing on those trials where mentalizing was most likely to occur—that is, same-speaker deliberation trials—did we find anticipatory (pre-naming) evoked activity (ERF analysis), but this was only for brain areas typically linked to episodic retrieval, linguistic predictions, and conflict anticipation, and not for areas typically involved in mentalizing. Importantly, episodic recall of speaker-specific representations is not identical to common ground, as episodic representations can be shared without also being known to be shared (Shintel and Keysar 2007). That is, representations may become activated via the basic memory retrieval principle of encoding specificity rather than through any process that genuinely reflects processing of the speaker's experience, such as produced by mentalizing (Barr et al. 2014).

It is important to emphasize that selecting this particular subset of same-speaker deliberation trials was most favorable for finding anticipatory mentalizing activity; if these trials do not show anticipation of the speaker's mental states in the TPJ that has been primarily associated with perspective taking, then no other trial type is more likely to do so. Thus, looking back at the 2 accounts we contrasted in Introduction, the results of our analyses support the conclusion that anticipating specific speakers' referential behavior based on mentalizing in relation to previously established common ground may not be a spontaneous, default process. In contrast, the default process seems to be more egocentric with anticipation only relying on episodic retrieval of visual and linguistic associations without any inference of the speaker's current mental states. The latter appears to be mainly engaged "on demand" once a pragmatic violation has been established and a deliberate decision has been made to account for it. Moreover, this evidence suggesting on-demand engagement of mentalizing has been obtained in the ecologically valid context of a realistic communicative interaction with live interlocutors.

Although the pattern of results was reassuringly consistent across our varied analyses, it is important to point out once again that the segregation of functional brain modules and the links between brain areas and function are based on the current state of the literature and must therefore be regarded as hypothetical and exploratory at the current stage. Future studies are necessary to corroborate these findings, yet we believe that the current results and interpretations will provide valuable constraints and hypotheses for future research. It should also be noted that our results were obtained with a novel, socially interactive paradigm that is quite different from most neurocognitive paradigms employed to date, which means that there is no clear evidentiary basis on which we could have generated more confirmatory-style predictions. Therefore, future research is needed to confirm the results of the present study, using similar or even more realistic paradigms. Further research is also needed to establish the generality of these results beyond the communicative scenario that we have investigated. It could well be the case that in certain scenarios mentalizing is indeed engaged more spontaneously, for example, in conversation with children who are not expected to adhere to common ground or to be able to fully engage in mentalizing themselves. Finally, compared with natural conversation, the present study still used a somewhat repetitive task, which was necessary at the current stage of our research for maintaining sufficient experimental control and statistical power. Still, the interactive nature of the paradigm should have been sufficient to have motivated participants to remain focused throughout the experiment. Our study could be a stepping stone toward even more naturalism, along a path that might eventually allow neuroimaging to tackle the unrestrained nature of real conversation.

## Conclusions

In the current MEG study, we employed an innovative experimental paradigm that combined an initial phase of live conversational interaction with a confederate speaker and a subsequent test phase of prerecorded speech from either the same or another speaker. Naturalistic negotiation of referential precedents in the interactive phase was sometimes followed by a referential mismatch in the test phase. The critical condition was when the same speaker produced a mismatch, requiring participants to engage in mentalizing in order to judge whether the speaker was still referring to the same object.

Based on a substantial body of previous research relating specific cognitive functions to certain brain areas, our results for theta oscillations, theta sources, theta phase couplings and evoked responses consistently indicate that brain areas typically involved in language, vision, episodic working memory and mentalizing were dynamically and jointly involved in resolving conflict after encountering a mismatching reference by the same speaker (but not another speaker). However, we found more robust mentalizing effects in the post-naming than in the pre-naming interval, suggesting that on a majority of trials, participants engaged in mentalizing only in response to a perceived violation. Only when focusing on those trials where mentalizing was most likely to occur did we find any anticipatory (pre-naming) activity, but this activity was confined to brain areas typically linked to episodic retrieval, linguistic predictions, and conflict anticipation, and did not include areas typically involved in mentalizing. Importantly, episodic recall of speaker-specific representations is not identical to common ground, as episodic representations can be shared without also being known to be shared (Shintel and Keysar 2007).

In conclusion, default processing of utterances that violate common ground seems to be quite egocentric at first, with anticipation relying primarily on episodic retrieval of visual and linguistic associations without any inference about the speaker's current mental states. The latter appears to be mainly engaged "on demand" once a pragmatic violation has been established and a deliberate decision has been made to account for it.

## Supplementary Material

Supplementary material can be found at: http://www.cercor.oxfordjournals.org/.

## Funding

This work was supported by the Netherlands Organisation for Scientific Research (Rubicon Fellowship, 446-10-020 to S.B.), the European Research Council (Advanced Grant INTERACT #269484 to Stephen Levinson [S.B.]), and the Economic and Social Research Council/Medical Research Council (RES-060-25-0010 to S.G. and RES-000-22-4325 to K.K.). Funding to pay the Open Access publication charges for this article was provided by the Economic and Social Research Council (ESRC, UK) via the University of Glasgow.

## Supplementary Material

Supplementary Data

## References

[BHU116C1] Aarts E Roelofs A van Turennout M 2008 Anticipatory activity in anterior cingulate cortex can be independent of conflict and error likelihood. J Neurosci. 28:4671–4678. 18448644 10.1523/JNEUROSCI.4400-07.2008 PMC6670453

[BHU116C2] Atique B Erb M Gharabaghi A Grodd W Anders S 2011 Task-specific activity and connectivity within the mentalizing network during emotion and intention mentalizing. Neuroimage. 55:1899–1911. 21172444 10.1016/j.neuroimage.2010.12.036

[BHU116C3] Baddeley A 2000 The episodic buffer: a new component of working memory? Trends Cogn Sci. 4:417–423. 11058819 10.1016/s1364-6613(00)01538-2

[BHU116C4] Barr DJ 2014 Perspective taking and its impostors: four patterns of deception. In: Holtgraves T, editor. Oxford Handbook of Language and Social Psychology. New York: Oxford University Press.

[BHU116C5] Barr DJ Jackson L Phillips I 2014 Using a voice to put a name to a face: the psycholinguistics of proper name comprehension. J Exp Psychol Gen. 143:404–413. 23398179 10.1037/a0031813

[BHU116C6] Bastiaansen MC Linden Mvd Keurs Mt Dijkstra T Hagoort P 2005 Theta responses are involved in lexical-semantic retrieval during language processing. J Cognit Neurosci. 17:530–541. 15814011 10.1162/0898929053279469

[BHU116C7] Bastiaansen M van Berkum JJ Hagoort P 2002 Syntactic processing modulates the θ rhythm of the human EEG. NeuroImage. 17:1479–1492. 12414287 10.1006/nimg.2002.1275

[BHU116C8] Brennan SE Clark HH 1996 Conceptual pacts and lexical choice in conversation. J Experim Psychol Learn Mem Cognit. 22:1482. 10.1037//0278-7393.22.6.1482 8921603

[BHU116C9] Castellanos NP Makarov VA 2006 Recovering EEG brain signals: artifact suppression with wavelet enhanced independent component analysis. J Neurosci Methods. 158:300–312. 16828877 10.1016/j.jneumeth.2006.05.033

[BHU116C10] Clark HH Brennan SE 1991 Grounding in communication. Perspect Soci Shared Cognit. 13:127–149.

[BHU116C11] Clark HH Marshall CR 1981 Definite reference and mutual knowledge. In: Joshe AK Webber BL Sag IA, editors. Elements of Discourse Understanding. Cambridge: Cambridge University Press p. 10–61.

[BHU116C12] Corbetta M Shulman GL 2002 Control of goal-directed and stimulus-driven attention in the brain. Nat Rev Neuroscience. 3:201–215. 11994752 10.1038/nrn755

[BHU116C13] Duff MC Brown-Schmidt S 2012 The hippocampus and the flexible use and processing of language. Front Hum Neurosci. 6. 10.3389/fnhum.2012.00069 PMC3319917 22493573

[BHU116C14] Dumontheil I Küster O Apperly IA Blakemore S-J 2010 Taking perspective into account in a communicative task. NeuroImage. 52:1574–1583. 20510369 10.1016/j.neuroimage.2010.05.056

[BHU116C15] Epstein R Kanwisher N 1998 A cortical representation of the local visual environment. Nature. 392:598–601. 9560155 10.1038/33402

[BHU116C16] Federmeier KD 2007 Thinking ahead: the role and roots of prediction in language comprehension. Psychophysiology. 44:491–505. 17521377 10.1111/j.1469-8986.2007.00531.x PMC2712632

[BHU116C17] Ferreira VS Dell GS 2000 Effect of ambiguity and lexical availability on syntactic and lexical production. Cognitive Psychol. 40:296–340. 10.1006/cogp.1999.0730 10888342

[BHU116C18] Frith CD Frith U 2006 The neural basis of mentalizing. Neuron. 50:531–534. 16701204 10.1016/j.neuron.2006.05.001

[BHU116C19] Fuentemilla L Penny WD Cashdollar N Bunzeck N Düzel E 2010 Theta-coupled periodic replay in working memory. Curr Biol. 20:606–612. 20303266 10.1016/j.cub.2010.01.057 PMC2856918

[BHU116C20] Gardner H Brownell HH Wapner W Michelow D 1983 Missing the point: the role of the right hemisphere in the processing of complex linguistic materials. In: Perecman E, editor. Cognitive Processing in the Right Hemisphere. New York: Academic Press. p. 169–191.

[BHU116C21] Giraud A-L Poeppel D 2012 Cortical oscillations and speech processing: emerging computational principles and operations. Nat Neurosci. 15:511–517. 22426255 10.1038/nn.3063 PMC4461038

[BHU116C22] Grandke T 1983 Interpolation algorithms for discrete Fourier transforms of weighted signals. Instrum Measur IEEE Transact. 32:350–355.

[BHU116C23] Gregory C Lough S Stone V Erzinclioglu S Martin L Baron-Cohen S Hodges JR 2002 Theory of mind in patients with frontal variant frontotemporal dementia and Alzheimer's disease: theoretical and practical implications. Brain. 125:752–764. 11912109 10.1093/brain/awf079

[BHU116C24] Groppe DM Urbach TP Kutas M 2011 Mass univariate analysis of event-related brain potentials/fields I: a critical tutorial review. Psychophysiology. 48:1711–1725. 21895683 10.1111/j.1469-8986.2011.01273.x PMC4060794

[BHU116C25] Gross J Baillet S Barnes GR Henson RN Hillebrand A Jensen O Jerbi K Litvak V Maess B Oostenveld R 2012 Good-practice for conducting and reporting MEG research. NeuroImage. 65:349–363. 23046981 10.1016/j.neuroimage.2012.10.001 PMC3925794

[BHU116C26] Gross J Kujala J Hämäläinen M Timmermann L Schnitzler A Salmelin R 2001 Dynamic imaging of coherent sources: studying neural interactions in the human brain. Proc Natl Acad Sci. 98:694–699. 11209067 10.1073/pnas.98.2.694 PMC14650

[BHU116C27] Gross J Schmitz F Schnitzler I Kessler K Shapiro K Hommel B Schnitzler A 2004 Modulation of long-range neural synchrony reflects temporal limitations of visual attention in humans. Proc Natl Acad Sci USA. 101:13050–13055. 15328408 10.1073/pnas.0404944101 PMC516515

[BHU116C28] Hagoort P Hald L Bastiaansen M Petersson KM 2004 Integration of word meaning and world knowledge in language comprehension. Science. 304:438–441. 15031438 10.1126/science.1095455

[BHU116C29] Hari R Kujala MV 2009 Brain basis of human social interaction: from concepts to brain imaging. Physiol Rev. 89:453–479. 19342612 10.1152/physrev.00041.2007

[BHU116C30] Horton WS Gerrig RJ 2005 The impact of memory demands on audience design during language production. Cognition. 96:127–142. 15925573 10.1016/j.cognition.2004.07.001

[BHU116C31] Imaizumi S Mori K Kiritani S Kawashima R Sugiura M Fukuda H Itoh K Kato T Nakamura A Hatano K 1997 Vocal identification of speaker and emotion activates different brain regions. Neuroreport. 8:2809–2812. 9295122 10.1097/00001756-199708180-00031

[BHU116C32] Jensen O Colgin LL 2007 Cross-frequency coupling between neuronal oscillations. Trends Cognit Sci. 11:267–269. 17548233 10.1016/j.tics.2007.05.003

[BHU116C33] Kessler K Biermann-Ruben K Jonas M Roman Siebner H Bäumer T Münchau A Schnitzler A 2006 Investigating the human mirror neuron system by means of cortical synchronization during the imitation of biological movements. NeuroImage. 33:227–238. 16876435 10.1016/j.neuroimage.2006.06.014

[BHU116C34] Kessler K Kiefer M 2005 Disturbing visual working memory: electrophysiological evidence for a role of the prefrontal cortex in recovery from interference. Cereb Cortex. 15:1075–1087. 15563725 10.1093/cercor/bhh208

[BHU116C35] Keysar B Lin S Barr DJ 2003 Limits on theory of mind use in adults. Cognition. 89:25–41. 12893123 10.1016/s0010-0277(03)00064-7

[BHU116C36] Kronmüller E Barr DJ 2007 Perspective-free pragmatics: Broken precedents and the recovery-from-preemption hypothesis. J Mem Lang. 56:436–455.

[BHU116C37] Lewis D 1979 Scorekeeping in a language game. J Philosophical Logic. 8:339–359.

[BHU116C38] Lin S Keysar B Epley N 2010 Reflexively mindblind: using theory of mind to interpret behavior requires effortful attention. J Experim Soc Psychol. 46:551–556.

[BHU116C39] Marini A Carlomagno S Caltagirone C Nocentini U 2005 The role played by the right hemisphere in the organization of complex textual structures. Brain Lang. 93:46–54. 15766767 10.1016/j.bandl.2004.08.002

[BHU116C40] Maris E Oostenveld R 2007 Nonparametric statistical testing of EEG-and MEG-data. J Neurosci Methods. 164:177–190. 17517438 10.1016/j.jneumeth.2007.03.024

[BHU116C41] Mars RB Sallet J Schüffelgen U Jbabdi S Toni I Rushworth MF 2012 Connectivity-based subdivisions of the human right “temporoparietal junction area”: evidence for different areas participating in different cortical networks. Cerebral Cortex. 22:1894–1903. 21955921 10.1093/cercor/bhr268

[BHU116C42] Matthews D Lieven E Tomasello M 2010 What's in a manner of speaking? Children's sensitivity to partner-specific referential precedents. Development Psychol. 46:749. 10.1037/a0019657 20604599

[BHU116C43] Metzing C Brennan SE 2003 When conceptual pacts are broken: partner-specific effects on the comprehension of referring expressions. J Mem Lang. 49:201–213.

[BHU116C44] Nilsen ES Graham SA 2009 The relations between children's communicative perspective-taking and executive functioning. Cognit Psychol. 58:220–249. 18809176 10.1016/j.cogpsych.2008.07.002

[BHU116C45] Nolte G 2003 The magnetic lead field theorem in the quasi-static approximation and its use for magnetoencephalography forward calculation in realistic volume conductors. Phys Med Biol. 48:3637. 14680264 10.1088/0031-9155/48/22/002

[BHU116C46] Noordzij ML Newman-Norlund SE De Ruiter JP Hagoort P Levinson SC Toni I Noordzij M Newman-Norlund S de Ruiter J Hagoort P 2009 Brain mechanisms underlying human communication. Front Hum Neurosci. 3:14. 19668699 10.3389/neuro.09.014.2009 PMC2722906

[BHU116C47] Olsen RK Nichols EA Chen J Hunt JF Glover GH Gabrieli JDE Wagner AD 2009 Performance-related sustained and anticipatory activity in human medial temporal lobe during delayed match-to-sample. J Neurosc. 29:11880–11890. 10.1523/JNEUROSCI.2245-09.2009 PMC2775810 19776274

[BHU116C48] Olson IR Moore KS Stark M Chatterjee A 2006 Visual working memory is impaired when the medial temporal lobe is damaged. J Cogn Neurosci. 18:1087–1097. 16839283 10.1162/jocn.2006.18.7.1087

[BHU116C49] Oostenveld R Fries P Maris E Schoffelen J-M 2011 FieldTrip: open source software for advanced analysis of MEG, EEG, and invasive electrophysiological data. Comput Intellig Neurosci. 2011:1. 10.1155/2011/156869 PMC3021840 21253357

[BHU116C50] Pfurtscheller G Lopes da Silva F 1999 Event-related EEG/MEG synchronization and desynchronization: basic principles. Clin Neurophysiol. 110:1842–1857. 10576479 10.1016/s1388-2457(99)00141-8

[BHU116C51] Pickering MJ Garrod S 2004 Toward a mechanistic psychology of dialogue. Behavior Brain Sci. 27:169–189. 10.1017/s0140525x04000056 15595235

[BHU116C52] Rankin KP Salazar A Gorno-Tempini ML Sollberger M Wilson SM Pavlic D Stanley CM Glenn S Weiner MW Miller BL 2009 Detecting sarcasm from paralinguistic cues: anatomic and cognitive correlates in neurodegenerative disease. Neuroimage. 47:2005–2015. 19501175 10.1016/j.neuroimage.2009.05.077 PMC2720152

[BHU116C53] Redcay E Dodell-Feder D Pearrow MJ Mavros PL Kleiner M Gabrieli JD Saxe R 2010 Live face-to-face interaction during fMRI: a new tool for social cognitive neuroscience. NeuroImage. 50:1639–1647. 20096792 10.1016/j.neuroimage.2010.01.052 PMC2849986

[BHU116C54] Roβnagel C 2000 Cognitive load and perspective-taking: applying the automatic-controlled distinction to verbal communication. Eur J Soc Psychol. 30:429–445.

[BHU116C55] Sakai K Passingham RE 2004 Prefrontal selection and medial temporal lobe reactivation in retrieval of short-term verbal information. Cereb Cortex. 14:914–921. 15115738 10.1093/cercor/bhh050

[BHU116C56] Samson D Apperly IA Chiavarino C Humphreys GW 2004 Left temporoparietal junction is necessary for representing someone else's belief. Nat Neurosci. 7:499–500. 15077111 10.1038/nn1223

[BHU116C57] Saxe R Powell LJ 2006 It's the thought that counts specific brain regions for one component of theory of mind. Psychol Sci. 17:692–699. 16913952 10.1111/j.1467-9280.2006.01768.x

[BHU116C58] Saxe R Wexler A 2005 Making sense of another mind: the role of the right temporo-parietal junction. Neuropsychologia. 43:1391–1399. 15936784 10.1016/j.neuropsychologia.2005.02.013

[BHU116C59] Schmidt D Krause B Weiss P Fink G Shah N Amorim M Müller H Berthoz A 2007 Visuospatial working memory and changes of the point of view in 3D space. Neuroimage. 36:955–968. 17493835 10.1016/j.neuroimage.2007.03.050

[BHU116C60] Shintel H Keysar B 2007 You said it before and you'll say it again: expectations of consistency in communication. J Experim Psychol Learn Mem Cognit. 33:357. 10.1037/0278-7393.33.2.357 17352617

[BHU116C61] Sohn MH Albert MV Jung K Carter CS Anderson JR 2007 Anticipation of conflict monitoring in the anterior cingulate cortex and the prefrontal cortex. Proc Natl Acad Sci. 104:10330–10334. 17563353 10.1073/pnas.0703225104 PMC1965513

[BHU116C62] Squire LR Zolamorgan S 1991 The medial temporal-lobe memory system. Science. 253:1380–1386. 1896849 10.1126/science.1896849

[BHU116C63] Stalnaker RC 1987 Inquiry. Cambridge: MIT Press.

[BHU116C64] Sulpizio V Committeri G Lambrey S Berthoz A Galati G 2013 Selective role of lingual/parahippocampal gyrus and retrosplenial complex in spatial memory across viewpoint changes relative to the environmental reference frame. Behav Brain Res. 242:62–75. 23274842 10.1016/j.bbr.2012.12.031

[BHU116C65] Tourville JA Guenther FH 2011 The DIVA model: A neural theory of speech acquisition and production. Lang Cognit Process. 26:952–981. 23667281 10.1080/01690960903498424 PMC3650855

[BHU116C66] Van Berkum JJ Brown CM Hagoort P 1999 Early referential context effects in sentence processing: evidence from event-related brain potentials. J Mem Lang. 41:147–182.

[BHU116C67] Van Berkum JJ Brown CM Hagoort P Zwitserlood P 2003 Event-related brain potentials reflect discourse-referential ambiguity in spoken language comprehension. Psychophysiology. 40:235–248. 12820864 10.1111/1469-8986.00025

[BHU116C68] Van Overwalle F Baetens K 2009 Understanding others’ actions and goals by mirror and mentalizing systems: a meta-analysis. Neuroimage. 48:564–584. 19524046 10.1016/j.neuroimage.2009.06.009

[BHU116C69] Van Veen BD Buckley KM 1988 Beamforming: a versatile approach to spatial filtering. ASSP Magazine, IEEE. 5:4–24.

