# Supplementary material for: Conversational Interaction in the Scanner: Mentalizing during Language Processing as Revealed by MEG
Source: Cereb Cortex. 2014 Jun 5;25(9):3219–34. doi: 10.1093/cercor/bhu116 (PMC4537451; doi:10.1093/cercor/bhu116)
Supplement: Supplementary Data [file supp_25_9_3219__index.html]

Conversational Interaction in the Scanner: Mentalizing during Language Processing as Revealed by MEG — Conversational Interaction in the Scanner: Mentalizing during Language Processing as Revealed by MEG — Supplementary Data 

# Conversational Interaction in the Scanner: Mentalizing during Language Processing as Revealed by MEG

## Supplementary Data

Supplementary Data

**Files in this Data Supplement:**

- Supplementary Data - Docx file
- Supplementary Table 1 - docx file
- Supplementary Table 2 - docx file
- Supplementary Table 3 - docx file
- Supplementary Table 4 - docx file
