# Supplementary material for: Conversational Interaction in the Scanner: Mentalizing during Language Processing as Revealed by MEG
Source: Cereb Cortex. 2014 Jun 5;25(9):3219–34. doi: 10.1093/cercor/bhu116 (PMC4537451; doi:10.1093/cercor/bhu116)

**SUPPLEMENTARY MATERIAL**

**Figure Captions**

Figure S1. Panel A: Time-Frequency representations (TFR) of effects in alpha (9-13 Hz) between about 200 and 400 ms, only found in the comparison between precedent mismatch versus no precedent for the same speaker (left column; p = .009), but not for the different speaker (right column). Channels showing significant effects in a representative time window (300-350 ms) are indicated by black dots (top left). Underneath, power values, relative to the baseline time window, are presented per condition and for differences between conditions for one channel, indicated by a white circle (top left). Panel B: Time-frequency representations of effects in gamma (66-78 Hz) between about 50 and 450 ms, only in the comparison between precedent mismatch deliberation versus no precedent trials for the same speaker in an analysis between 0 and 500 ms (p = .028). Channels showing significant effects in a representative time window (150-200 ms) are indicated by black dots (top). Underneath, power values, relative to the baseline window, are presented for the two conditions for two significant channels indicated by white circles (top). Black squares indicate the location of the effects in time and frequencies.

Figure S2. Sensor level ERF. Comparisons for precedent mismatch versus no precedent for same speaker (left column) and different speaker (right column). Topographical plots show planar gradient values per condition. A significant cluster was found for the same speaker precedent mismatch versus no precedent conditions (p = .02) between 318 and 454 ms. For the comparison precedent mismatch versus no precedent for the different speaker, a significant cluster was found (p = .048) between 300 and 415 ms.Significant clusters (black asterisks) at a representative time are indicated for both contrasts separately on top of t-values (bottom). Waveforms are plotted for one representative channel, indicated by the white circle. Figure 7 shows additional sensor level ERF results for deliberation trials.


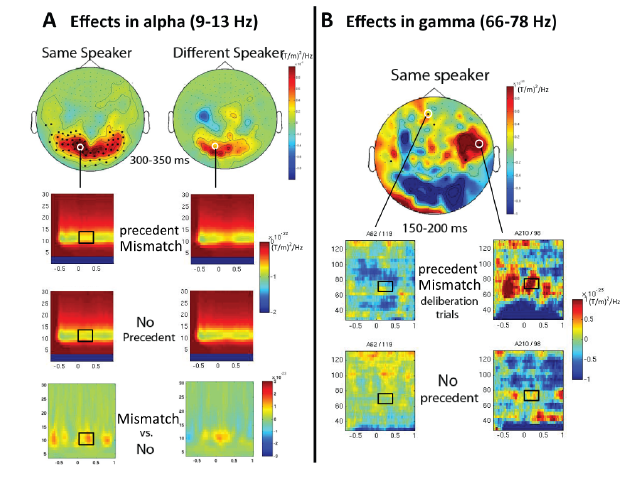

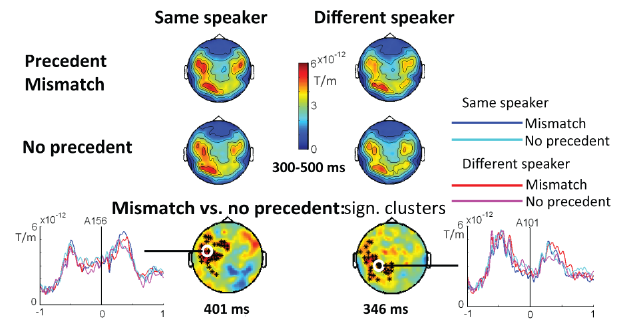

Supplement: Supplementary Data [file supp_bhu116_bhu116supp.docx]
