# Supplementary material for: Conversational Interaction in the Scanner: Mentalizing during Language Processing as Revealed by MEG
Source: Cereb Cortex. 2014 Jun 5;25(9):3219–34. doi: 10.1093/cercor/bhu116 (PMC4537451; doi:10.1093/cercor/bhu116)
Supplement: Supplementary Data [file supp_bhu116_bhu116supp_table1.docx]

Table S1 (related to Figure 4). Labels, Brodmann areas, and coordinates for local maxima (T-values) of the sources identified in Figure 4.

| **Panel A**:  Area label | BA | Coordinates of local maxima | | |  |  | **Panel B:**  Area label | BA | Coordinates of local maxima | | |
| --- | --- | --- | --- | --- | --- | --- | --- | --- | --- | --- | --- |
| Left PMC | 6 | -24 | 20 | 58 |  |  | Left SM1 | 6/4/3 | -36 | -4 | 66 |
| Left SM1 | 4,6 | -58 | -2 | 56 |  |  | Left vmPFC | 10 | -24 | 72 | 6 |
| Left vmPFC | 10 | -24 | 72 | 2 |  |  | Left latPFC | 46/45 | -56 | 42 | 10 |
| Left latPFC | 46,45 | -50 | 46 | 10 |  |  | Left/bilateral vmPFC | 25/11 | 2 | 14 | -22 |
| Left TC (TP) | 22 | -64 | -4 | 6 |  |  | Left TC (TP) | 38 | -30 | 6 | -36 |
| Right SM1 | 3 | 68 | -16 | 38 |  |  | Right latPFC | 10 | 40 | 62 | 10 |
| Right PPC | 7 | 28 | -54 | 44 |  |  | Right PPC | 7 | 26 | -82 | 48 |
| Right TPJ | 22,39,40 | 60 | -54 | 16 |  |  | Right TPJ | 40,3 | 68 | -46 | 24 |
| Right PC | 7 | 12 | -44 | 48 |  |  | (two maxima) | 37,21 | 64 | -62 | 2 |
| Right PHG/MTL | 36 | 28 | -36 | -10 |  |  | Right PHG/MTL | 36 | 24 | -38 | -10 |
| Right OCC | 18,17 | 26 | -88 | 2 |  |  | Right PC | 7 | 12 | -48 | 44 |
|  |  |  |  |  |  |  | Right TP | 21,38 | 40 | 24 | -46 |
