# Supplementary material for: Conversational Interaction in the Scanner: Mentalizing during Language Processing as Revealed by MEG
Source: Cereb Cortex. 2014 Jun 5;25(9):3219–34. doi: 10.1093/cercor/bhu116 (PMC4537451; doi:10.1093/cercor/bhu116)
Supplement: Supplementary Data [file supp_bhu116_bhu116supp_table2.docx]

Table S2 (related to Figure 5). Labels, Brodmann areas, and coordinates

for local maxima (T-values) of the sources identified in Figure 5.

| Area label | BA | Coordinates of local maxima | | |
| --- | --- | --- | --- | --- |
| Left (P)MC | 6 | -54 | -4 | 50 |
| bilateral vmPFC | 11 | 0 | 34 | -34 |
| Left TC/ITG | 21/22/20 | -72 | -20 | -6 |
| Left TP | 20,38 | -42 | 0 | -28 |
| Right TP | 21,38 | 40 | 24 | -50 |
| Right PPC | 7 | 42 | -54 | 64 |
| Right TPJ | 40 | 60 | -54 | 24 |
| Right SM1 | 40,2,1,3 | 72 | -24 | 28 |
| Right PC | 7 | 16 | -62 | 52 |
| Right PHG/MTL | 37,19,36 | 36 | -40 | -8 |
| Left OCC | 19 | -22 | -86 | 28 |
| Right OCC | 17 | 12 | -68 | 28 |
