# Supplementary material for: Conversational Interaction in the Scanner: Mentalizing during Language Processing as Revealed by MEG
Source: Cereb Cortex. 2014 Jun 5;25(9):3219–34. doi: 10.1093/cercor/bhu116 (PMC4537451; doi:10.1093/cercor/bhu116)
Supplement: Supplementary Data [file supp_bhu116_bhu116supp_table3.docx]

Table S3 (related to Figure 6). Labels, Brodmann areas, and coordinates for local maxima (T-values) of the "seed areas" and their coherent sources in Figure 6.

| Seed area | Coherent area | BA | Coordinates of local maxima | | |
| --- | --- | --- | --- | --- | --- |
| Left TP |  | 38,21 | -40 | 10 | -22 |
|  | Left vmPFC | 10,11 | -36 | 62 | -8 |
|  | Left latPFC | 9 | -42 | 36 | 34 |
|  | Left latPFC | 8 | -42 | 20 | 46 |
| Left MTC |  | 22,21 | -60 | -10 | 4 |
|  | Right latPFC | 13 | 36 | 6 | 20 |
|  | Right latPFC | 9 | 38 | 8 | 24 |
|  | vmPFC | 10 | -2 | 52 | -2 |
|  | Caudal ACC | 32,24 | 2 | 12 | 38 |
|  | Left PHG | 19,30 | -10 | -46 | -6 |
| Right PHG |  | 36 | 28 | -36 | -12 |
|  | Left mTC | 20 | -62 | -24 | -22 |
|  | Left SMA | 6 | -36 | 22 | 58 |
|  | Right latPFC | 10 | 42 | 60 | 30 |
|  | Left TPJ | 39,40 | -44 | -54 | 30 |
| Right TPJ |  | 40 | 62 | -32 | 30 |
|  | Left vmPFC | 11 | -30 | 54 | -20 |
|  | Right mTC (reduced) | 21 | 70 | -40 | -12 |
|  | Right PMC (reduced) | 6,9 | 56 | 8 | 36 |
| Left latPFC |  | 46,10 | -38 | 40 | 4 |
|  | Right mTC (reduced) | 21,20 | 68 | -22 | -16 |
|  | Right latPFC | 10 | 42 | 54 | 10 |
