# Supplementary material for: Conversational Interaction in the Scanner: Mentalizing during Language Processing as Revealed by MEG
Source: Cereb Cortex. 2014 Jun 5;25(9):3219–34. doi: 10.1093/cercor/bhu116 (PMC4537451; doi:10.1093/cercor/bhu116)
Supplement: Supplementary Data [file supp_bhu116_bhu116supp_table4.docx]

Table S4 (related to Figure 8). Labels, Brodmann areas, and coordinates for local maxima (T-values) of the sources identified in Figure 8.

| **Panel A (early)**:  Area label | BA | Coordinates of local maxima | | |  |  | **Panel B (late):**  Area label | BA | Coordinates of local maxima | | |
| --- | --- | --- | --- | --- | --- | --- | --- | --- | --- | --- | --- |
| Right dlPFC | 46 | 50 | 36 | 12 |  |  | Left SM1 | 4/6/3 | -30 | -20 | 58 |
| bilateral ACC | 32/24 | 6 | 10 | 20 |  |  | bilateral ACC | 32/24 | -6 | 22 | 26 |
| Right TC | 22 | 60 | -32 | 4 |  |  | Left PHG (MTL) | 19 | -36 | -48 | -4 |
| Right OTC | 19 | 38 | -68 | -6 |  |  | Left TPJ | 40 | -68 | -42 | 34 |
| Right POC | 19/7 | 26 | -74 | 34 |  |  | Left vmPFC | 11 | -32 | 36 | -22 |
| bilateral OCC | 18 | -10 | -72 | 16 |  |  | Left vlPFC | 47/11 | -50 | 42 | -18 |
| Left POC | 18/31 | -8 | -72 | 16 |  |  | Right vlPFC | 47 | -46 | 36 | -8 |
| Left OTC | 37/21 | -56 | -66 | 10 |  |  | Right SMA | 6/8 | 8 | -5 | 68 |
| Left PHG (MTL) | 30 | -28 | 54 | 2 |  |  | Left TP | 38 | 46 | 22 | -18 |
|  |  |  |  |  |  |  | Left PMC | 6/8/9 | -68 | 12 | 44 |
